# Plant extracts and phytochemicals in canine and feline mammary cancer models: current evidence and comparative perspectives

**DOI:** 10.1007/s11259-026-11336-8

**Published:** 2026-06-17

**Authors:** Iason-Spyridon Patergiannakis, Ioannis S. Pappas

**Affiliations:** Laboratory of Pharmacology and Toxicology, Faculty of Veterinary Medicine, University of Thessaly, 43100 Karditsa, Thessaly Greece

**Keywords:** Mammary tumor, Cancer, Canine, Phytochemical, Natural product, Feline

## Abstract

Mammary tumors are among the most common neoplasms in dogs and cats, and malignant forms are clinically important in both species. Surgery remains the main treatment approach and may be combined with other treatment modalities, but advanced cases are often associated with recurrence and limited responsiveness to conventional therapies. Phytochemicals and plant extracts have received attention as potential adjuvant agents that can modulate pathways associated with oncogenesis and treatment resistance. Natural products are often accessible and relatively inexpensive, and their perception as well-tolerated agents may increase owner acceptance. Some plant extracts and phytochemicals, including *Euphorbia royleana* extract*,* celastrol, and homoharringtonine have shown potent preclinical activity in canine mammary tumor cell lines and xenograft models, but data for feline mammary tumors are still limited. However, their safety and efficacy depend on multiple factors such as the compound, dose, formulation, and species. Further studies are required for clinical translation, as species-specific pharmacokinetics significantly affect efficacy and safety. This review summarizes current knowledge on plant extracts and phytochemicals in canine and feline mammary tumors, focusing on their preclinical evidence, limitations, and future translational challenges in veterinary oncology.

**Supplementary Information:**

The online version contains supplementary material available at https://doi.org/10.1007/s11259-026-11336-8.

## Introduction

Mammary cancer is one of the most common neoplasms in dogs and cats. Canine mammary tumors (CMTs) represent more than 50% of neoplasms in intact bitches, whereas in males, they account for less than 1% of neoplasms (Vazquez et al. 2023; Miranda et al. 2025). Feline mammary tumors (FMTs) account for 17% of tumors in queens, and fewer than 5% of FMTs occur in male cats (Morris 2013; Gameiro et al. 2021c). More than 80% of FMT cases are malignant, with metastases occurring usually in the lymph nodes and lungs (Gameiro et al. 2021c). Malignant CMTs represent approximately 40–60% of mammary tumors (Rodríguez et al. 2022; Vazquez et al. 2023). A number of factors, including age, breed, steroidal hormones (mainly the balance between androgens, estrogens, and progesterone), reproductive status, and hereditary gene mutations, are associated with tumorigenesis in mammary tissue (da Silva et al. 2023; Singla et al. 2023; Monteiro et al. 2025a). Moreover, the type of diet, dog size, housing, and obesity may also affect the development of CMT (da Silva et al. 2023). Emerging evidence implicates oral, gut and mammary microbiota in the development of mammary tumors (Zheng et al. 2022; Singla et al. 2023). Differences between the microbiota of healthy mammary tissue and tumors have been observed in humans and dogs (Zheng et al. 2022; Furuta 2024), although the mechanisms and clinical relevance remain unclear and are under investigation (Feng et al. 2024a; Nazeer et al. 2025).

In both species, spaying at a young age decreases the risk of developing mammary cancer due to changes in hormonal balance. The primary treatment choice for most CMT and FMT cases is surgical excision (Morris 2013; Vazquez et al. 2023). In certain cases, treatment may also include chemotherapeutic drugs, while radiation therapy and electrochemotherapy can be added (Cassali et al. 2024). Other remedies, such as immunotherapy, hormonal therapy, DNA vaccines, and the use of oncolytic viruses, are being studied (Sánchez et al. 2018; Valdivia et al. 2021; Glikin and Finocchiaro 2025). Chemotherapy is introduced in veterinary patients with malignant mammary tumors. Although the treatment efficiency is low and the recurrence rate is high, the main goal is to improve quality of life (Elliot and Mayer 2009). A different approach is considered in advanced-stage cancer with metastasis and inflammatory mammary carcinoma (IMC) (Valdivia et al. 2021), where radiation therapy, cyclooxygenase-2 (COX-2) inhibitors, tyrosine kinase inhibitors and metronomic chemotherapy are considered (Alonso-Miguel et al. 2022).

Current cancer therapies present various limitations, such as limited accessibility to certain remedies (Kidd 2008), high costs with uncertain results and adverse effects that clients may not be willing to be involved in (Stephens 2019; Biegel et al. 2022). Moreover, both tumors and potent cancer therapies can result in cachexia (Munteanu et al. 2024). Considering also the multifactorial nature of mammary cancer, plant-derived products have gained attention as potential adjuvants with antiproliferative, pro-apoptotic, and antimetastatic effects in both in vitro and in vivo models.

Phytomedicine has been applied traditionally in both human and veterinary medicine for many years (Fougère and Wynn 2006; Chunarkar-Patil et al. 2024). Medicinal plants are widely accessible and may exert multimodal biological effects (El-Saadony et al. 2025). Plants contain a wide variety of bioactive secondary metabolites with potential medicinal properties. As a result, plants can be used either as a source of these compounds, which can be used as precursors for drug synthesis and discovery, or as extracts, teas, foods, etc. Different phytochemicals exhibit anticancer properties and have been studied for use in cancer treatment (Naeem et al. 2022). Various medicinal plants and phytochemicals have exhibited anticancer properties against breast cancer in in vitro and in vivo models (Singla et al. 2023). Due to the variety of phytochemicals found in natural products, their potential anticancer activity is attributed to various mechanisms (Rabelo et al. 2021). Thus, their relevance as adjuvant candidates lies in their ability to act on multiple tumor-related processes simultaneously, including proliferation, apoptosis, invasion, angiogenesis, and treatment resistance, which are important aspects in mammary cancer treatment (Naeem et al. 2022; Al Khalily et al. 2025). Although phytochemicals have been widely investigated in human breast cancer (HBC), no structured review has focused specifically on their effects on canine and feline mammary tumors.

Because direct evidence linking plant extracts and phytochemicals to microbiome-mediated tumor suppression in CMTs and FMTs remains limited, this review focuses primarily on their direct anticancer and adjuvant effects. It provides a structured overview of the available in vitro and in vivo evidence in these species, with emphasis on comparative oncology insights, therapeutic potential, and current gaps in the field.

## Materials and methods

This review aimed to identify original research articles evaluating the effects of plant extracts and phytochemicals on canine or feline mammary cancer, using in vitro or in vivo models. A structured literature search was conducted in PubMed and Scopus for studies published between January 2010 and July 2025. The following keywords and Boolean operators (“AND”, “OR”) were used in various combinations: *“Canine mammary tumor”, “Canine mammary cancer”, “Canine breast cancer”, “Canine mammary carcinoma cell line”, “CMT-U27”, “CMT-U309”, “Feline mammary tumor”, “Feline mammary cancer”, “Feline breast cancer”, “Feline mammary carcinoma cell line”, “FCTp”, “FMCm”, “herbal extract”, “plant extract”, “phytochemical”, “natural product”* (Online Resource 1).

Beyond database searches, additional relevant studies were identified through citation tracking of included articles and targeted searches in Google Scholar. Although the primary inclusion period was 2010–2025, older foundational studies were also considered when identified through citations.

### Exclusion criteria

To maintain the focus on plant-derived products, the review excluded synthetic drugs inspired by natural compounds (e.g., aspirin, metformin, atorvastatin, abemaciclib, alpelisib, rivoceranib), as well as semi-synthetic phytochemical derivatives. Natural products of non-plant origin (e.g., microbial metabolites like rapamycin, fungal statins, or marine-derived carotenoids such as fucoxanthin) were also excluded. Non-phytochemical therapies, including 5-aminolevulinic acid in photodynamic therapy, were not considered.

## Plant-derived products studied

Only a few plant-derived products have been studied for possible anticancer activity in CMTs, and very little data are available in FMT models. These products include both plant extracts and isolated phytochemicals. As these two categories differ in terms of composition, standardization, and mechanistic interpretation, they are discussed separately below.

### Plant extracts

Plant extracts, often derived from plants with traditional medicinal uses, have been evaluated in vitro on established cell lines or primary tumor cultures and, in some cases, in vivo or in clinical veterinary settings. Reported effects include direct cytotoxicity, inhibition of proliferation and migration, induction of apoptosis or autophagy, modulation of tumor-related signaling pathways, and antiangiogenic effects. However, the potency and selectivity of these extracts vary considerably. In many cases, their activity remains below the thresholds considered physiologically relevant for potential therapeutic development, and no corresponding extract-based study was identified in FMT models.

Table 1 summarizes the plant extracts evaluated in canine mammary tumor research, detailing their experimental models, primary findings, and reported half-maximal inhibitory concentration (*IC*_*50*_) values where available. This compilation highlights both promising candidates for further investigation and extracts whose activity may be limited in a therapeutic context.

**Table 1:** Summary of plant extracts investigated for their effects on CMT models, as no corresponding study on FMT models was identified. The table includes the type of model, cytotoxic potency, key findings, and relevant notes. Extracts with reported *IC*_50_ values below 30 μg/mL have their potency data highlighted in bold, as this threshold is commonly used in the natural-products literature to indicate promising in vitro cytotoxic activity. The standardized commercial products of *Punica granatum* and *Piper nigrum*, although slightly above this threshold, are also highlighted because their defined composition may reduce batch-to-batch variability and improve reproducibility; however, their potency should be interpreted more cautiously than that of extracts clearly meeting the threshold. *Plantago asiatica* polysaccharides are also noted because, despite lacking direct cytotoxicity, they showed synergistic antitumor effects in murine models when combined with taxol. Reported constituents are mentioned only where explicitly described for the tested preparation. In the less potent ethanolic aerial-part extract of *Calotropis procera*, the reported constituents included isorhamnetin-3-O-hexose-deoxyhexoside, kaempferol 3-O-hexoside, kaempferol-3-O-hexose-deoxyhexoside, quercetin-3-O-hexoside riojaposide A, isorhamnetin, calotropin, coroglaucigenin, calotropagenin, roseosides I and II, adynerin, and roseoside. Commercial extracts of *Camellia sinensis*, *Curcuma longa*, *P. nigrum*, *P. granatum*, and *Rosmarinus officinalis* were reported to contain 45.76% epigallocatechin gallate (EGCG), 87.59% curcuminoids, 95.02% piperine, 35.06% punicalagins, and 66.9% carnosic acid, respectively. *Typhonium flagelliforme* extract was reported to contain flavonoids, steroids, and tannins, but not triterpenoids, alkaloids, or saponins. For *Viscum album*, the veterinary post-surgical study used a commercial mistletoe preparation (ISCADOR® P), for which product information describes a fermented aqueous extract of *V. album* from specific host trees, with sodium chloride and water for injection as excipients, however, detailed phytochemical characterization of the preparation was not provided

Plant extracts (common name)	Experimental model(s)	Key findings	*IC*_50_/Potency	Notes	Evidence level	References
***Calotropis procera*** (giant milkweed/swallow-wort/apple of Sodom)	Primary CMT culture; CF41.Mg cells	Cytotoxicity; altered cell morphology; reduced proliferation	Ethyl acetate of aerial parts of the plant was the most potent fraction on primary culture: 140 μg/mL **Ethanolic extract of the roots on CF41.Mg cells:** **9 μg/mL**	Two extracts obtained from an ethanolic extract may not reflect full phytochemical profile. Ethanolic extract was more selective towards CMT cells than doxorubicin. The root extract exhibited strong cytotoxic effect	In vitro only	(Rabelo et al. 2021; Vahidi et al. 2021)
***Camellia sinensis*** (green tea) Product name: Green tea extract (Naturex)	CMT-12 cells	Cytotoxic	**20.4 μg/mL**	Promising cytotoxic effect	In vitro only	(Levine et al. 2016)
***Curcuma longa*** (turmeric) Product name: Turmeric extract (Naturex)	CMT-12 cells	Cytotoxic; Combined with chemotherapy: antagonistic effect at low concentrations, additive effects at > 3.1 μg/mL	**9.1 μg/mL**	Synergistic effects with Rosemary extract;	In vitro only	(Levine et al. 2016, 2017)
***Dendrophthoe pentandra*** (clove mistletoe)	MCM-B2 cells (benign mixed mammary tumor)	*IC*_50_ not reached at ≤ 125 μg/mL	> 125 μg/mL	Limited cytotoxic potency	In vitro only	(Elsyana et al. 2016)
***Euphorbia royleana*** (Sullus spurge/Royle’s spurge/Churee)	CMT1 & MPG cells; mice xenograft (CMT1)	Cytotoxic & cytostatic; induced autophagy; decreased p53 expression; Synergistic activity with the autophagy inhibitor Bafilomycin A1; inhibited tumor growth and increased necrosis in vivo	**Hexane extract on MPG: 3.5 μg/mL** **On CMT1: 4.5 μg/mL**	Ethanolic & hexane extracts tested. Hexane extract exhibited stronger cytotoxic activity on cell lines In vivo dosage: 10 mg/kg orally every 2–4 days	In vitro + xenograft	(Huang et al. 2020)
***Gynura procumbens*** (Longevity spinach/Scrambling gynura)	CHMp-13a & CHMp-5b cell lines	Cytotoxic, suppressed migration (scratch assay), increased apoptosis, decreased EGFR expression	Not determined up to a concentration 160 μg/mL	Almost reached 50% cell growth inhibition at 160 μg/mL at 28 h	In vitro only	(Jermnak et al. 2022)
***Piper nigrum*** (black pepper) Product name: VetPerine (Sabinsa)	CMT-12 cells	Mild cytotoxicity	**34.5 μg/mL**	Close to the proposed threshold for promising extracts	In vitro only	(Levine et al. 2016)
***Plantago asiatica*** Isolation of Plantain polysaccharide (PLP)	CIPp canine mammary tumor cells; CIPp xenograft in BALB/c mice;	No direct cytotoxicity on CIPp cells; promoted dendritic cell (DC) maturation and lymphocyte proliferation	**Not directly cytotoxic**	Antitumor effects are immune-mediated rather than direct; most effective when combined with tumor lysates or chemotherapy	In vitro + xenograft	(Gao et al. 2021)
***Punica granatum*** (pomegranate) Product name: Pomegranate extract [40% punicosides] (Polinat)	CMT-12 cells	Cytotoxic, but with limited potency Above the proposed potency threshold	**40.9 μg/mL**	Above the proposed threshold for promising extract cytotoxicity	In vitro only	(Levine et al. 2016)
***Rosmarinus officinalis*** (rosemary) Product name: Rosemary extract INOLENS70 (Vitiva)	CMT-12 cells	Cytotoxic; Combined with chemotherapy: antagonistic effect at low concentrations, additive effects at > 3.1 μg/mL	**13 μg/mL**	Synergistic with turmeric via JNK pathway; increased curcumin accumulation in cells	In vitro only	(Levine et al. 2016, 2017)
***Typhonium flagelliforme*** (Rodent tuber)	MCM-IPB-B3 cells (benign mixed tumor)	Growth inhibition; synergistic with canine interferons; antiangiogenic in rabbit endothelial cells and in ovo	Did not reach 50% cell growth inhibition at 120 μg/mL	Ethanolic extract tested	In vitro only	(Priosoeryanto et al. 2020)
***Viscum album*** (European mistletoe)	Post-surgery dogs; UNESP-CM9 & UNESP-CM60 CMT lines	Post-surgery use: non-significant trend toward reduced tumor-related deaths (p = 0.07); in vitro cytotoxicity; delayed wound closure (scratch assay)	On UNESP-CM9: 3.11 μL/100 μL On UNESP-CM60: 2.99 μL/100 μL	Unknown plant mass used (final concentration) Synergistic effect with resveratrol	In vitro + Post-surgical veterinary use	(Biegel et al. 2017; Vaz et al. 2024)

**Abbreviations:** *CMT* canine mammary tumor, *DC* dendritic cells, *IC*_50_ half-maximal inhibitory concentration, *JNK* c-jun N-terminal kinase

**Fig. 1:**
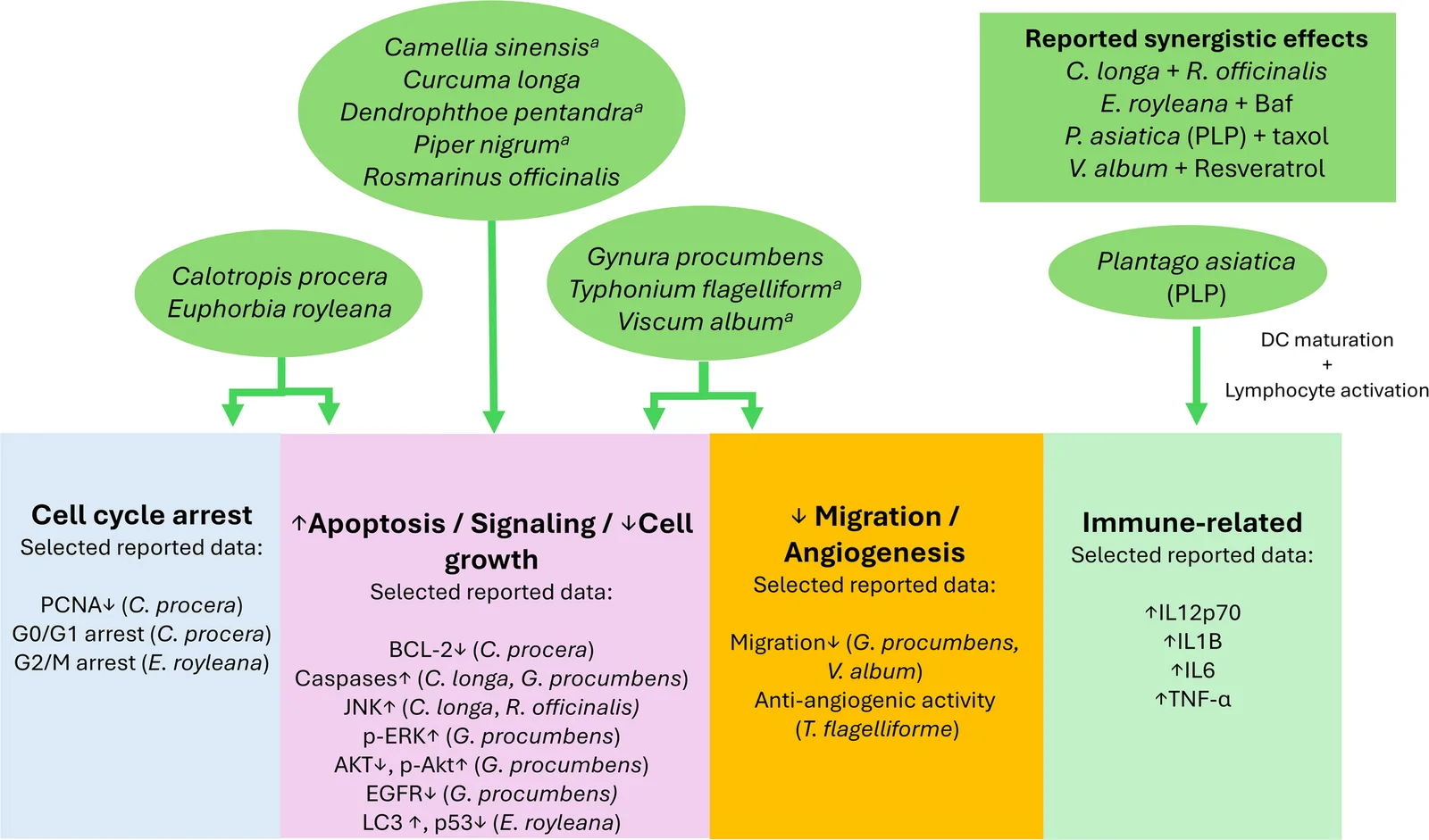
Comparative overview of plant extracts investigated in canine mammary tumor models. No studies evaluating extract effects in feline mammary tumor models were identified. Extracts are grouped according to the main biological effects reported in the original studies, including cell-cycle arrest, apoptosis/signaling/reduced cell growth, reduced migration/angiogenesis, and immune-related activity. Selected reported data are shown only where molecular or cellular readouts were explicitly evaluated. The box at the upper right summarizes reported synergistic effects. “a” Placement in the indicated category was based mainly on reported phenotypic effects, since specific mechanistic factors were not evaluated in detail in the original study. Absence of a listed factor should not be interpreted as absence of biological activity, but rather as lack of assessment or reporting in the corresponding study. **Abbreviations**: Baf, Bafilomycin A1; Bcl-2, B-cell lymphoma 2; EGFR, epidermal growth factor receptor**;** IL, interleukin; LC3, microtubule-associated light chain 3; PCNA, proliferating cell nuclear antigen; p-ERK, phosphorylated extracellular signal-regulated kinase; TNF-α, tumor necrosis factor-α

The biological activity of plant extracts likely reflects the combined contribution of multiple constituents, making it difficult to attribute the observed effects to a single compound. In some studies, the tested preparations were at least partially characterized and major constituents were reported, however, even in these cases, the overall activity is likely to remain multimodal. Isolated phytochemicals offer a more suitable framework for discussing mechanism of action and certain aspects of metabolism that can affect translational relevance. Owing to extract variability, the information on extract efficacy and composition discussed here is restricted to the studies summarized in Table 1. Plant extracts showed heterogeneous but mainly cytotoxic, antiproliferative, pro-apoptotic, antimigratory, antiangiogenic, or immune-modulating effects, whereas mechanistic evaluation was available only for a subset of studies. *Calotropis procera* extract reduced proliferation, induced G0/G1 arrest, decreased proliferating cell nuclear antigen (PCNA) and downregulated B-cell lymphoma 2 (Bcl-2) (Rabelo et al. 2021; Vahidi et al. 2021). Extracts of *Camellia sinensis, Curcuma longa*, *Piper nigrum*, *Punica granatum* and *Rosmarinus officinalis*, were cytotoxic in CMT-12 cells, *C. longa* being the most potent and exhibiting synergistic activity with *R. officinalis* (Levine et al. 2016). *C. longa* treatment resulted in caspase activation and apoptosis, while the combination with *R. officinalis* enhanced this effect and activated c-jun N-terminal kinase (JNK) (Levine et al. 2017). *Euphorbia royleana* extract induced cytotoxicity, autophagy, p53 downregulation, and upregulation of microtubule-associated light chain 3 (LC3), necrosis and G2/M arrest. Moreover, *E. royleana* extract exhibited synergy with autophagy inhibitor, bafilomycin A1 (Huang et al. 2020). *Gynura procumbens* demonstrated antiproliferative and antimigratory effects, increased caspase 3/7 activity reduced epidermal growth factor receptor (EGFR) expression, altered AKT/ERK-related signaling, and affected phosphatase and tensin homolog (PTEN) and twist family bHLH transcription factor 1 (TWIST) expression (Jermnak et al. 2022). *Plantago asiatica* polysaccharides showed no marked direct cytotoxicity but promoted dendritic-cell maturation, cytokine secretion, lymphocyte activation, and taxol-associated antitumor effects, indicating an immune-mediated mode of action (Gao et al. 2021). For *Viscum album*, *Typhonium flagelliforme*, and *Dendrophthoe pentandra*, only limited mechanistic information was available (Elsyana et al. 2016; Biegel et al. 2017; Priosoeryanto et al. 2020; Vaz et al. 2024). The reported effects are summarized in Fig. 1.

### Isolated phytochemicals

A variety of isolated phytochemicals have exhibited anticancer activity against CMT. These compounds exert their effects through a variety of mechanisms. Table 2 summarizes the main isolated phytochemicals reported in the literature, detailing their natural sources, mechanisms of action, and documented effects in CMT and FMT models.

**Table 2:** Summary of phytochemicals investigated in CMT and FMT models, including the type of model, anticancer effects, cytotoxic potency, key findings and relevant notes. Notably, only one study was identified evaluating the potential activity of a phytochemical (curcumin) in FMT models. Phytochemicals with reported IC_50_ values below 10 μM are considered more promising candidates for further investigation. However, compounds with values close to this benchmark should not necessarily be excluded based on potency alone. Diallyl disulfide exhibited an IC_50_ of 10.9 μM and was retained among the compounds of interest. In addition, some agents with higher IC_50_ values have demonstrated antitumor activity in in vivo models and may also warrant further study. Agents considered more relevant for follow-up investigation are highlighted in bold in the potency column

Compound (*Source*)	Experimental model(s)	Key findings	*IC*_50_/potency	Notes	Evidence level	References
**Aconitine-type diterpenoids (aconitine, aconitine linoleate, indaconitine, mesaconitine)** (*Aconitum* spp.)	CMT-7364 cells (triple-negative CMT)	Aconitine linoleate: inhibited proliferation and arrested cell cycle; induced apoptosis	Aconitine and indaconitine: > 400 μM **Aconitine linoleate: 8.14 μM** Mesaconitine > 50 μM	Aconitine exhibits neurotoxic and cardiotoxic effects	In vitro only	(Zhang et al. 2023)
**Benzyl isothiocyanate (BITC)** (cruciferous vegetables)	CIPp & CMT-7364 cells; CIPp xenograft in mice	Decreased growth, migration, invasion; increased apoptosis; cell-cycle arrest; inhibited tumor growth in vivo	CMT-7364: ~ 40% cell viability at 25 μM (48 h); **CIPp: ~ 50% viability at 6.25 μM**	In vivo activity confirmed in xenograft In vivo dosage: 20 mg/kg/day intraperitoneal (IP)	In vitro + xenograft	(Cheng et al. 2020)
**Berberine** **(BBR)** (*Berberis vulgaris* and others)	CF41.Mg & CF33 cells	Inhibited proliferation, increased early apoptosis; decreased Wnt/β-catenin signaling	> 200 μM (CF41.Mg); > 40 μM (CF33)	Antioxidant on normal cells, pro-oxidant on tumor cells	In vitro only	(Sefidabi et al. 2017; Sammarco et al. 2023a)
**Cannabidiol (CBD)** (*Cannabis sativa*)	IPC366 and CF41.Mg	Inhibited cell viability and colony formation for up to 20 days; increased apoptosis; reduced migration and invasion	> 50 μM for CBD-Nem in both cell lines 20 μΜ CBD-E induced ~ 40–60% inhibition in both cell lines	Nanoemulsion-based formulation to improve aqueous solubility; CBD-Nem reduced viability more selectively in CF41.Mg than in non-neoplastic MDCK cells	In vitro only	(Medina et al. 2025)
**Celastrol** (*Tripterygium wilfordii*)	CMT-7364 cells, CIPp cells	Potent cytotoxicity; inhibited proliferation, migration, invasion; increased apoptosis; cell-cycle arrest	** ~ 1 μM (CMT-7364), 0.97 μM (CIPp)**	—	In vitro only	(Ou et al. 2022)
**Curcumin** (*Curcuma longa*) Lipocurc**™** (SignPath Pharma Inc.) /Curcumin	Lipocurc: CMT12Q2 & CMT27 cells; in vivo in 2 dogs with primary mammary carcinoma, with evaluable outcome data available for only one dog Curcumin: primary canine mammary cells – derived from simple carcinoma (SC) and squamous cell carcinoma (SCC)	Lipocurc IV form: decreased *IC*_50_ in CMT12Q2, increased in CMT27; no tumor size change in treated dog; Curcumin: induced apoptosis in both SC and SCC cells; induced cycle arrest in SC cells but not in SCC; changes in cellular morphology	CMT12Q2: Lipocurc (3.3 μg/mL) vs curcumin (6.5 μg/mL) CMT27: Lipocurc (14.4 μg/mL) vs curcumin (11.2 μg/mL) **0.5 μM Curcumin decreased viability by 56.52% and 40.64% on SC and SCC cells, respectively**	Poor oral bioavailability; Lipocurc improves delivery In vivo dosage: 10 mg/kg infusion over 8 h intravenous (IV) SC were more sensitive than SCC doses ≥ 20 mg/kg → hemolysis in beagles. Danger of acute hemolysis upon bolus administration of Lipocurc	In vitro + in vivo pilot veterinary clinical trial	(Withers et al. 2018; Turna et al. 2022)
	Two distinct feline mammary gland tumor carcinoma primary cell cultures (C1 and C2)	In both cell lines: Curcumin decreased cell viability; increased apoptosis; it induced morphological changes in cells (vacuolization, round shape, apoptotic bodies, and necrotic morphology) In C2 it also affected significantly the cell cycle at higher concentrations	In C1 cell viability was reduced by 44% at the highest concentration tested (1 mM) In C2 cell viability was decreased by 42% and 62% with the 0.1 mM and 0.5 mM treatment, respectively	The concentrations tested were higher than in other studies; however, these findings suggest measurable in vitro activity of curcumin in aggressive feline mammary gland tumor carcinoma primary cultures	In vitro only	(Deveci Ozkan et al. 2021)
**Esculetin (6,7-dihydroxycoumarin)** (*Aesculus hippocastanum*, *Artemisia capillaris*, etc.)	CMT-U27 and CF41.Mg cells	CMT-U27: 0.125 mM → ~ 40% viability; suppressed migration at 0.25 mM; increased apoptosis by tenfold;	CMT-U27: 0.125 mM → ~ 40% viability;	Less potent on CF41.Mg	In vitro only	(Choi et al. 2023)
**Genistein** (*Genista tinctoria*)	CMT-U27 and CF41.Mg	Inhibited cell proliferation, viability; induced apoptosis and cell-cycle arrest	CMT-U27: 100 μM reduced viability by 72% at 24 h CF41.Mg: 80 μM reduced viability by ~ 20%	Synergistic anticancer effect with ERB-041	In vitro only	(Yoo et al. 2024; Jang et al. 2025)
**Homoharringtonine** **(HHT)** (*Cephalotaxus* spp.)	CMT-U27 cells	Inhibited proliferation; Decreased viability and colony formation, migration and invasion; induced apoptosis;	**8.25 nM at 48 h**	Regulates AKT/mTOR pathway	In vitro only	(Zhang et al. 2024)
**Indole-3-carbinol (I3C)** (cruciferous vegetables)	Mouse xenograft of canine inflammatory mammary carcinoma	Reduced tumor growth & ulceration, increased apoptosis, hormonal changes; Increased liver metastases (significant) and distant metastases (non-significant)	**N/A**	Potential pro-metastatic risk noted in vivo In vivo dosage: 150 mg/kg/day	Xenograft only	(Martín-Ruiz et al. 2018)
**Isorhamnetin** **(ISO)** *(Ginkgo biloba* and other plants)	CMT-U27 cells; U27 −/− (PD-L1 knockout) cells; U27 xenograft nude mice	inhibited migration and invasion; disrupted mitochondrial integrity; induced apoptosis; inhibited tumor growth	≈ 128 μM	In vivo tumor growth suppressed by 46% (50 mg/kg, TID for 9 days) in U27 xenograft mice	In vitro + xenograft	(Mei et al. 2024)
**Matrine** (*Sophora* spp.)	CHMm & CHMp canine mammary tumor cells; primary mammary epithelial cells	Inhibited proliferation; induced autophagy and apoptosis, mitochondrial swelling	No marked toxicity reported up to 250 μg/mL in normal mammary epithelial cells; inhibitory effects in tumor cells at 62.5–250 μg/mL	Proposed direct target: BTF3; reported to bind and stabilize protein; downregulates *Btf3* gene expression	In vitro only	(Feng et al. 2024b)
**Methyl gallate (MG)** *(various plants, e.g. Mangifera indica*, *Terminalia myriocarpa*)	CMT-U27 and CF41.Mg cells; CMT xenograft mouse model; canine endothelial cells	Inhibited cell viability and migration; induced apoptosis with increased cleaved caspase-3; in vivo: suppressed tumor growth, reduced vessel density, increased apoptosis;	**N/A**	Inhibited migration and tube formation in endothelial cells In vivo dosage 40 mg/kg	In vitro + xenograft	(Choi et al. 2024)
**Organosulfur compounds** (*Allium sativum*, garlic)	CMT-13 cells	Fat-soluble OSCs inhibited cell growth; water-soluble OSCs inactive	**Diallyl disulfide: 10.9 μM**	Included diallyl sulfide, disulfide, trisulfide	In vitro only	(Sundaram and Milner 1993)
**Palmatine** (*Annona glabra*, *Berberis* spp., *Tinospora cordifolia*, etc.)	CMT-U27 cells; xenograft	Inhibited tumor growth; increased cell death; decreased angiogenesis, metastasis; Cell morphological changes, cytotoxic and apoptotic effects	**50 μM induced 53.4% cell death**	in vitro and in vivo effects inhibited PI3K/AKT/mTOR in vivo dosage: 50 mg/kg/day IP for 21 days	In vitro + xenograft	(Yoo et al. 2023)
**Resveratrol** **(RES)** (*Vitis vinifera*, others)	UNESP-CM9, UNESP-CM60 NK pretreatment before treating CHMm cells; xenograft	NK cell pretreatment with RES enhances the effects of NK on CHMm cells RES induces cell death and decreases cell migration	UNESP-CM9 (281.6 μM) UNESP-CM60 (105.5 μM)	Immunomodulatory effects	In vitro + xenograft	(Vaz et al. 2024; Zhu et al. 2024)
**Toosendanin** **(TSN)** (*Melia toosendan*)	CMT-U27 cells; xenograft in mice	Morphological changes; inhibited DNA synthesis; decreased migration and invasion; increased apoptosis; inhibited tumor growth in vivo	**19.37 μM after** **48 h**	In vivo dosage: 1 mg/kg IP every 2 days for 20 days	In vitro + xenograft	(Yang et al. 2023)
**Zerumbone** **(ZER)** (*Zingiber zerumbet*)	Metastatic CMT cells (ER⁻/PR⁺/HER2⁺)	GI₅₀: 11.4 μM (free) → 4.3 μM (lipid carrier); increased antiproliferative effect with encapsulation; induced cell death	**4.3–11.4 μM**	Nanocarrier delivery enhanced potency	In vitro only	(Foong et al. 2018)

**Abbreviations:** *BTF3* basic transcription factor 3, *CBD-E* CBD dissolved in ethanol, *CBD-Nem* CBD loaded into oil-in-water nanoemulsions, *CMT* canine mammary tumor, *ER* estrogen receptor, *HER2* human epidermal growth factor receptor 2, *IC*50 half-maximal inhibitory concentration, *IP* intraperitoneal, *IV* intravenous, *mTOR* mechanistic target of rapamycin, *NK* natural killer, *PD-L1* programmed cell death ligand 1, *PI3K* phosphoinositide 3-kinase, *SC* simple carcinoma, *SCC* squamous cell carcinoma, *Wnt* wingless-type MMTV integration site family

### Translational considerations

A wide range of concentrations of plant extracts and phytochemicals have been reported to affect mammary cancer cells and tumors. Findings from in vitro studies are inherently difficult to translate directly into clinical relevance. Although all available data may be informative, some products are more potent than others. In the literature a frequently used threshold for promising cytotoxic activity of crude plant extracts is an *IC*_50_ below 30 μg/mL (Ahmad et al. 2010; Stefanowicz-Hajduk et al. 2021; Canga et al. 2022; Charles-Okhe et al. 2022). For isolated compounds a commonly used threshold is an *IC*_50_ below 10 μM (Mbaveng et al. 2017; Lee et al. 2020; Feitosa et al. 2024). While these thresholds cannot determine the absolute translational value of a given phytochemical or plant extract, they provide a practical way for interpreting the relative potency of the studied agents and distinguishing more promising candidates from those requiring more cautious interpretation in future studies. Results derived from in vivo experiments, including xenografts and clinical studies, generally provide a stronger basis for translational interpretation than in vitro findings alone, although their relevance varies according to the model used.

Among the more promising extracts discussed in this review are *Calotropis procera* (root extract), *Camellia sinensis*, *Curcuma longa, Euphorbia royleana, Rosmarinus officinalis,* and *Plantago asiatica*. Although *Plantago asiatica* PLP extract did not exhibit direct cytotoxic activity in vitro, it demonstrated synergistic antitumor effects in murine xenograft models when combined with taxol, with associated tumor reduction, stable body weight, and decreased splenic mass. However, a major limitation of botanical extracts is their compositional variability, which may depend on geographic origin, environmental conditions, and harvesting time (Pant et al. 2021; Sun et al. 2025). Extraction conditions, including solvent, temperature, pH, particle size and extraction time, may further alter the phytochemical profile of the final extract (Sun et al. 2025). This variability may be reduced in standardized commercial products such as Polinat (pomegranate extract) (*IC*_50_ = 40.9 μg/mL), Rosemary extract (*IC*_50_ = 13 μg/mL), and Sabinsa (black pepper extract) (*IC*_50_ = 34.5 μg/mL) (Levine et al. 2016). Consequently, although Polinat, and Sabinsa achieve *IC*_50_ values slightly above the threshold of 30 μg/mL, their standardized composition may support consideration for further evaluation.

Isolated phytochemicals reduce the issue of batch-to-batch variability that affects botanical extracts, although they may not reproduce the broader multimodal activity of whole extracts. In the present review, compounds considered more relevant for further investigation included those achieving *IC*_50_ values below 10 μM in at least one mammary tumor cell line, such as aconitine linoleate, benzyl isothiocyanate, celastrol, homoharringtonine, and zerumbone. While aconitine linoleate exhibits potent activity in a triple negative canine mammary tumor cell line, there is known cardiotoxic and neurotoxic activity related to aconitine (Zhang et al. 2023). Curcumin was also retained among the compounds of interest because, although an *IC*_50_ was not determined in one canine primary-cell study, it reduced viability by 56.52% in simple carcinoma cells at 0.5 μM (Withers et al. 2018; Turna et al. 2022). However, its translational relevance remains constrained by potential induced hemolysis and the limited in vivo response observed in the single dog for which evaluable data were available (Withers et al. 2018). Diallyl disulfide was also included because its reported *IC*_50_ (10.9 μM) was close to the 10 μM benchmark. In addition, compounds with higher *IC*_50_ values but supported by in vivo antitumor activity, including indole-3-carbinol (I3C), toosendanin, methyl gallate, and palmatine, may also warrant further investigation. It is worth noting that although I3C reduced tumor growth and ulceration in murine xenograft models of IMC, it was also associated with a significant increase in liver metastasis (Martín-Ruiz et al. 2018).

## Phytochemical metabolism and mechanisms of action

### Aconitine

Aconitine, a natural alkaloid, undergoes extensive phase I metabolism, primarily mediated by cytochrome P450 enzymes (CYP2C9, CYP2C8, CYP3A4, CYP3A5, CYP1A1, and CYP1A2) and carboxylesterases. Its metabolites are eliminated mainly via the urinary route (Zhao et al. 2024). In breast cancer, aconitine linoleate has been reported to inhibit topoisomerase II activity (Luan et al. 2022). Aconitine suppresses proliferation via inhibition of phosphoinositide 3-kinase/Protein kinase B (PI3K/Akt) pathway, reduces invasion through modulation of the transforming growth factor-β (TGF-β)/suppressor of mothers against decapentaplegic (Smad) pathway and downregulates both nuclear factor kappa-light-chain-enhancer of activated B cells (NF-κB) and receptor activator of nuclear factor-kappa B (RANK) expression (Li et al. 2022).

### Benzyl isothiocyanate (BITC)

Benzyl isothiocyanate (BITC) is a well-studied natural isothiocyanate (ITC) found in cruciferous vegetables (cabbage, broccoli, etc.). ITCs conjugate with glutathione (GSH) both enzymatically and spontaneously (Lamy et al. 2011). Specifically, the enzymatic systems involved in BITC metabolism include glutathione-S-transferase (GST), glutamyl transpeptidase (GGT), and CYP enzymes, and a major metabolite in dogs is hippuric acid (Brüsewitz et al. 1977; Lamy et al. 2011). BITC is a lipophilic molecule that has low solubility in water (Dinh et al. 2021). Most of BITC is excreted in the urine in dogs, while only a small amount is excreted in the feces (Brüsewitz et al. 1977). BITC activity has been studied in various types of cancer, and particularly in breast cancer (Dinh et al. 2021). BITC induces apoptosis in breast cancer cells via mitochondria-dependent pathways involving activation of caspases, modulation of Bcl-2 family proteins, and downregulation of survivin and XIAP (X-linked inhibitor of apoptosis). It also affects cell cycle parameters, metastasis, and angiogenesis by modulating MAPK (mitogen activated protein kinase) and wingless-type MMTV integration site family (Wnt)/β-catenin signaling, inhibiting vascular endothelial growth factor (VEGF) expression, and promoting autophagy (Dinh et al. 2021). In CIPp-induced mouse xenografts, BITC-induced apoptosis and cell-cycle arrest were associated with increased Bax expression and reduced levels of Bcl-2, cyclin-dependent kinase 1 (CDK1), and cyclin B1 (Cheng et al. 2020).

### Berberine (BBR)

Berberine (BBR) is unstable and has low solubility in water. However, its hydrochloride form overcomes these challenges and becomes stable and soluble in water (Qi et al. 2025). BBR quasi-irreversibly inhibits CYP2D6 (Qi et al. 2025). After oral administration of BBR, only a small amount is absorbed (less than 1%). BBR is metabolized through processes such as demethylation and glucuronidation in a variety of phase I and phase II metabolites, which retain some activity. The formation of phase I metabolites is primarily mediated by CYP2D6 and CYP1A2 (Khoshandam et al. 2022). The main enzyme contributing to the formation of phase II metabolites is uridine 5’-diphospho-glucuronosyltransferase (UDPGT) (Feng et al. 2018), although sulfotransferases (SULTs) and catechol-O-methyltransferase (COMT) are also involved in phase II metabolism (Khoshandam et al. 2022). Oral BBR administration also affects the intestinal microbiota in dogs, promoting health benefits (Feng et al. 2018). In breast cancer, BBR induces cell-cycle arrest and apoptosis and suppresses proliferation, migration, and drug resistance through multiple mechanisms. These include modulation of Wnt/β-catenin and adenosine monophosphate-activated kinase (AMPK) pathways, inhibition of protein kinase C-α (PKC-α)/matrix metalloproteinase (MMP) signaling, and activation of tumor suppressors such as p53, p21, and p27. BBR also affects epigenetic and microRNA regulation, and reduces hypoxia-inducible factor-1α (HIF-1α) and P-glycoprotein (P-gp) expression, contributing to its antiproliferative and chemosensitizing effects (Zhong et al. 2022). In studies involving CMT cells, inhibition of Wnt/β-catenin and activation of the Hippo pathway were reported (Sammarco et al. 2023a). Berberine could potentially have prolonged action in cats, as they lack major UDPGT enzymes, therefore, caution would be warranted if this compound is evaluated in feline mammary cancer.

### Cannabidiol (CBD)

Cannabidiol (CBD) is a well-studied non-psychoactive phytocannabinoid with poor aqueous solubility and limited oral bioavailability, prompting the investigation of alternative formulations and routes of administration. In dogs and cats, CBD shows variable pharmacokinetics, with reported half-lives depending on species, dose, feeding status, and formulation (Di Salvo et al. 2023; Rozental et al. 2023). After absorption, CBD undergoes hepatic metabolism and is excreted mainly in feces and partly in urine, while its metabolism through CYP and UDPGT enzymes raises the possibility of pharmacokinetic interactions (Beers et al. 2021; Czigle et al. 2023; Kamutchat et al. 2025). In breast cancer, CBD has mainly been studied in human models, where it has exhibited cytotoxicity, activation of peroxisome proliferator-activated receptor gamma (PPARγ), inhibition of PI3K/AKT/mTOR, MMP2, breast cancer resistance protein (BCRP) and MAPK, downregulation of inhibitor of DNA binding 1 (ID1), Bcl-2, XIAP and cyclin D1, upregulation of p53 and, suppression of proliferation and metastasis (García-Morales et al. 2023; Esmaeli et al. 2025; Çifçi et al. 2026). It has also exhibited synergistic effects with doxorubicin and taxol in vitro (Esmaeli et al. 2025). In canine mammary cancer cell lines CBD exhibited cytotoxicity, with increased apoptosis and altered cell-cycle distribution, inhibition of migration, invasion and long-term colony formation (Medina et al. 2025). However, specific pathway-level mechanisms have not been defined in canine mammary carcinoma models.

### Celastrol

Celastrol or tripterine exhibits poor absorption and bioavailability when administered orally to beagle dogs, and the concentration–time profile fits the pharmacokinetic model of one compartment (Wang et al. 2024). Celastrol is an inhibitor of cytochrome P450 (CYP) enzymes, inhibiting various enzymes of this family, and it also inhibits UDPGT (Zhou et al. 2025a). Thus, it should be used with caution in drug combinations, as it could aggravate toxicity (Wang et al. 2024). Moreover, in rats, celastrol downregulated BCRP and Multidrug resistance-associated protein (MRP) (Zhou et al. 2025a). In breast cancer models, celastrol reduces migration and invasion, attenuates inflammation, and induces mitochondria-mediated apoptosis via Bcl-2 downregulation and Bax upregulation. It modulates PI3K/Akt signaling, promotes mechanistic target of rapamycin (mTOR) degradation, suppresses NF-κB and MEK/ERK pathways, and downregulates MMPs and pro-inflammatory interleukins (Wang et al. 2023a). In CIPp and CMT-7364 (a triple-negative cell line) canine mammary cancer cells, celastrol induced apoptosis via Bax, caspase-3, and caspase-9 upregulation, and Bcl-2, NF-κB, and phosphorylated p65 downregulation, which is consistent with findings in other models (Ou et al. 2022; Wang et al. 2023a). It also causes cell-cycle arrest by increasing p21 and p27 levels and reducing cyclin D1 expression (Ou et al. 2022).

### Curcumin (Curc)

Curcumin (Curc) is a phytochemical found in turmeric with promising anticancer activity. The limitations of curcumin include its poor absorption from the gut and its fast metabolism (Revalde et al. 2015). Lipocurc (SignPath Pharma Inc.) allows intravenous drug delivery, overcoming the poor bioavailability by oral administration (Withers et al. 2018). A study conducted in vivo in beagles revealed that Lipocurc doses of 20 mg/kg or more can result in dose-dependent hemolysis. Curcumin is rapidly metabolized or distributed to tissue lipids, and thus plasma levels remain relatively low (Helson et al. 2012; Matabudul et al. 2012). It has been found to inhibit various CYP enzymes, including those involved in steroidogenesis and metabolic enzymes (Larasati et al. 2018; Castaño et al. 2019; Dibaei et al. 2024). Curcumin modulates multiple oncogenic pathways in breast cancer, including inhibition of Wnt/β-catenin, PI3K/Akt, EGFR, and NF-κB signaling, VEGF, IL-8 leading to reduced proliferation and angiogenesis and increased apoptosis, senescence, and autophagy, the latter being supported by increased LC3 (Akkoç et al. 2015; Mayo et al. 2024). It also upregulates tumor-suppressive miRNAs, while downregulates oncogenic miRNAs and inhibits cyclins and CDKs. In triple-negative breast cancer (TNBC), curcumin suppresses proliferation, invasion, and migration via modulation of the hedgehog (Hh)/glioma-associated oncogene homolog-1 (Gli1) pathway (Mayo et al. 2024).

### Esculetin (6,7-dihydroxycoumarin)

Esculetin, a simple coumarin, has low oral bioavailability due to extensive first-pass hepatic metabolism, primarily via glucuronidation mediated by UDPGT enzymes (Zhang et al. 2022b). Although absorption is low in beagle dogs, plant extracts containing esculetin, such as *Ledum palustre*, may include other bioactive compounds with similar pharmacological activities but different pharmacokinetic profiles (Wang et al. 2018b). In breast cancer, esculetin exerts apoptotic and cytotoxic effects, inhibits proliferation, and induces cell-cycle arrest through CDK1 and cyclin B1 downregulation, and p21, p53, caspase-3, caspase-9, and cytochrome c upregulation (Rezoan Hossain et al. 2024). In CMT cells, esculetin inhibited CDK4 and cyclin D1 and increased caspase-3 levels (Choi et al. 2023).

### Genistein

Genistein, an isoflavone, is rapidly absorbed and eliminated in beagle dogs. It is excreted predominantly via feces, with smaller amounts detected in urine (Zhou et al. 2003, 2005). Its metabolism occurs mainly through glucuronidation and sulfation, with minimal involvement of CYP enzymes, occurring in the intestine, liver, breast and prostate tissue. In mammary tissue, genistein is present primarily as genistein-7-O-glucuronide, and in humans, supplementation can result in tissue exposure levels sufficient to exert potential biological effects (Bolca et al. 2010; Yang et al. 2012). In breast cancer, genistein induces apoptosis through activation of the PPARγ pathway and caspase- and calpain-mediated mechanisms, while simultaneously reducing COX-2 activity and inflammation. It downregulates MMPs, upregulates p21, and decreases VEGF and TGF-β1. Genistein also promotes cell-cycle arrest and exerts antiproliferative effects by modulating the MAPK and PI3K/Akt pathways, inhibiting Polo-like kinase 1 (PLK1), and suppressing NF-κB signaling. Additional effects include the downregulation of Hypoxia inducible factor 1α (HIF-1α) and Hh/Gli1 pathways (Konstantinou et al. 2024). In CMT, it exerts its anticancer activity by modulating multiple pathways. It activates protein kinase R-like endoplasmic reticulum kinase (PERK)—activating transcription factor 4 (ATF4)—C/EBP homologous protein (CHOP) pathway, while suppressing inositol-requiring enzyme 1α (IRE1α)—X-box-binding protein 1 (XBP1) pathway. Genistein promotes caspase -mediated apoptosis, increasing Bax and decreasing Bcl-2 expression. It also downregulates estrogen receptors (ERα and ERβ) and inhibits PI3K/Akt/mTOR pathway, limiting proliferative signaling (Yoo et al. 2024; Jang et al. 2025).

### Homoharringtonine (HHT)

Homoharringtonine (HHT) in dogs is primarily cleared through tissue binding and metabolism, with elimination occurring mainly via the urinary route (Lu et al. 1988). In plasma, HHT undergoes hydrolysis by esterases, yielding inactive metabolites and cephalotaxine, whereas hepatic metabolism contributes minimally to its biotransformation (Solimando et al. 2013). Mechanistically, in breast cancer, HHT exerts its anticancer effects by targeting multiple pathways linked to apoptosis, survival and stemness. It downregulates Bcl-2, XIAP, survivin, suppresses miR-18a-3p, thereby inhibiting the AKT/mTOR pathway. HHT activates pro-apoptotic regulators such as Bax, caspase-3 and caspase-9. It also downregulates stemness-related markers, including octamer-binding transcription factor 4 (Oct4), CD44, SRY-box transcription factor 2 (Sox2), and nanog homeobox (Nanog), and decreases the proportion of breast cancer stem cells (BCSCs), (CD44^+^/CD24^−^). Additionally, it inhibits Hh/Gli1 pathway (Wang et al. 2025). In CMT, HHT downregulated Akt and mTOR gene expression, decreased p-AKT and p-mTOR protein levels, and induced apoptosis. TUNEL assays revealed karyopyknosis and nuclear fragmentation, while western blotting demonstrated upregulation of p53, Bax, cleaved caspase-3, and cleaved caspase-9 and downregulation of Bcl-2, resulting in an increased Bax/Bcl-2 ratio. These findings indicate that HHT inhibits proliferation, migration, and invasion of canine mammary carcinoma (CMC) cells through regulation of the AKT/mTOR pathway and induction of mitochondrial apoptosis (Zhang et al. 2024).

### Indole-3-carbinol (I3C)

In rats, I3C undergoes rapid elimination and acid-catalyzed conversion in the gut to diindolylmethane (DIM), a bioactive metabolite with neuroprotective (Ramakrishna et al. 2022) and anticancer properties, targeting cancer stem cells (CSCs) (Semov et al. 2012). These factors reduce the bioavailability of I3C. Both I3C and DIM are distributed to highly perfused organs (Ramakrishna et al. 2022). I3C induces several enzymes, including CYP (CYP1, CYP1A1 in the mammary gland, and CYP2B) and GSTs (Rogan 2006). I3C promotes DNA repair, induces cell-cycle arrest and apoptosis, inhibits cell migration, and modulates hormone receptor signaling. I3C has been shown to cause downregulation of CDK6 and activation of p53 (Brew et al. 2006). It disrupts NF-κB–dependent cell cycle progression, regulates the expression of DNA repair proteins, and inhibits WW domain-containing E3 ubiquitin protein ligase 1 (WWP1), thereby reactivating the tumor suppressor PTEN and suppressing PI3K/Akt-driven tumorigenesis (Centofanti et al. 2023). It causes the downregulation of ER-α and the upregulation of breast cancer gene1 (BRCA1) and E-cadherin (Meng et al. 2000a, b). In addition, I3C alters hormone metabolism by increasing the 2-hydroxyestrone/16α-hydroxyestrone ratio (Centofanti et al. 2023). In mouse xenograft models of canine IMC, I3C induced hormonal changes, reduced ulceration and tumor growth, and promoted apoptosis (Martín-Ruiz et al. 2018).

### Isorhamnetin (ISO)

Isorhamnetin (ISO) is the 3′-O-methylated metabolite of quercetin and can be produced by quercetin metabolism in the body (Tanaka et al. 2019). It undergoes further metabolism primarily through phase II conjugation, serving as a substrate for UDPGTs and being subject to deconjugation by β-glucuronidase and sulfatase enzymes (Chen et al. 2008; Tanaka et al. 2019). Like other flavonoids, ISO also interacts with drug-metabolizing enzymes, acting as an inhibitor of CYP1A2 and CYP2A6 activity (Bojić et al. 2019). In addition, BCRP, P-gp, and MRPs have been reported to be involved in its transport, with MRP2 appearing to play a particularly important role (Duan et al. 2014). ISO inhibits the Akt/mTOR/p70S6K signaling pathway and promotes mitochondria-dependent apoptosis in breast cancer cells. It downregulates the cyclin B1/CDK1 complex, suppresses the expression and activity of MMPs, and blocks the phosphorylation of p38, MAPK, and signal transducer and activator of transcription 3 (STAT3). ISO further upregulates Bax and caspases, while downregulating Bcl-2, thereby enhancing apoptosis. In addition, ISO has been found to reduce AMPK, and MEK/ERK signaling, and to decrease adhesion, migration and invasion (Rana et al. 2025). In CMT, ISO downregulated p-EGFR, p-STAT3, and programmed cell death 1 ligand 1 (PD-L1), with CRISPR/Cas9-mediated CD274 (PD-L1) knockout confirming that its antitumor effects were mediated through the EGFR–STAT3–PD-L1 signaling axis. In CMT-U27 xenograft mice, it increased caspase-3 and reduced Ki-67 and PD-L1 expression. Further mechanistic studies identified EGFR as a direct molecular target (Mei et al. 2024).

### Organosulfur compounds (Diallyl disulfide, DADS)

Diallyl disulfide (DADS) has limited oral bioavailability, due to its polarity, molecular weight, and first-pass metabolism. Owing to its lipophilicity, it is rapidly absorbed and distributed to lipid-rich tissues. DADS is metabolized primarily by CYP and sulfotransferases, and is excreted in urine (Zhou et al. 2025b). In breast cancer, DADS induces intrinsic apoptosis by modulating Bcl-2 family proteins, inhibits histone deacetylase (HDAC) activity, and upregulates miR-34a to suppress MAPK/ERK signaling. It has also been reported to inhibit tumor necrosis factor-α- (TNF-α), RAS-, and NF-κB-related signaling. It reduces BCSC growth and metastasis via Pyruvate kinase M2 (PKM2), CD44, AMPK, and β-catenin pathways, downregulates vimentin, p38 and MMP-9, while increases E-cadherin and reverses EMT, indicating both chemopreventive and antimetastatic potential (Mitra et al. 2022).

### Matrine (MT)

Matrine (MT) has a short half-life and acts as an inhibitor of the human organic cation transporter 3 (hOCT3), which plays a role in drug absorption, distribution, and elimination (Li et al. 2021a). It shows a low plasma protein-binding rate and has been reported to upregulate CYP enzymes such as CYP3A30, CYP2A6, CYP2B6, and CYP3A4 in hepatocytes (Gong et al. 2015; Li et al. 2021a). However, in vitro studies using rat liver microsomes demonstrated that matrine is not metabolized by CYP or UDPGT enzymes, suggesting limited biotransformation via conventional hepatic pathways (Yang et al. 2010). In breast cancer, MT exerts its antitumor effects through multiple signaling pathways. MT has been shown to induce cell-cycle arrest. Moreover, it inhibits AKT phosphorylation, enhances PTEN expression, and increases Let-7b miRNA, thereby influencing c-Myc, Ras, JAK/STAT3, and Wnt signaling. MT promotes apoptosis via PTEN upregulation and Bcl-2 and Bax modulation, miR-21 inhibition, suppression of mTOR, and blockade of the PI3K/Akt pathway. It also reduces p62 and increases LC3, suppresses VEGF, and downregulates NF-κB and MMPs, limiting angiogenesis and invasion. MT modulates immune responses by upregulating TGF-β, interleukin-6 (IL-6), and IL-10, but downregulating IL-2 and IFN-γ, and reverses drug resistance through downregulation of multi-drug resistance 1 (MDR1), P-gp, and MRP1 (Yang et al. 2025). In CMT, matrine is stabilized and bound directly to the basic transcription factor 3 (BTF3) protein, as confirmed by cellular thermal shift assay (CETSA) and molecular docking (binding site: Thr-89), and downregulates Btf3 gene expression over time (Feng et al. 2024b).

### Methyl gallate (MG)

Methyl gallate (MG) undergoes metabolism involving UDPGTs and β-glucuronidase (Liang et al. 2023). In breast cancer models, MG has been shown to modulate several signaling pathways. Transcriptomic pathway analysis predicted activation/upregulation of unfolded protein response (UPR) related pathways, and suppression of oncogenic and survival pathways through the downregulation of Akt, NF-κB, and mTOR in MCF-7 cells. Treatment with MG resulted in the downregulation of antiapoptotic proteins Bcl-2 and Bcl-xL, while pro-apoptotic mediators such as Bax, Bim, TP53, BAD, and PTEN were upregulated (Raut et al. 2025).

### Palmatine (PLT)

Palmatine (PLT) has low bioavailability, and in dogs, oral administration results in a maximal plasma concentration after 5 h (Huang et al. 2007; Tarabasz and Kukula-Koch 2020). PLT is a P-gp substrate, activating the transporter at low doses and weakly inhibiting it at relatively high concentrations (Long et al. 2019; Tarabasz and Kukula-Koch 2020). It modulates hepatic CYP activity, weakly inhibiting CYP2D6, CYP3A4, and CYP1A1, while activating CYP2C9 and CYP2C19, and acts as partial aryl hydrocarbon receptor (AhR) agonist. Metabolism occurs in the liver via hydroxylation, demethylation, and conjugation, and is excreted through bile, urine, and feces (Long et al. 2019; Tarabasz and Kukula-Koch 2020). In breast cancer, PLT suppresses MCF-7 cell proliferation by upregulating miR-200c and exhibits photosensitizing activity (Long et al. 2019). In CMT cells, PLT downregulated PI3K, AKT, mTOR, and PTEN expression while increasing phosphorylated PTEN (p-PTEN) levels. Furthermore, PLT inhibited angiogenesis, as evidenced by the reduced expression of vascular markers CD31 and α-smooth muscle actin (α-SMA) in CMT-U27 cells (Yoo et al. 2023).

### Resveratrol (RES)

Resveratrol (RES) exhibits low oral bioavailability in dogs, estimated at approximately 2%, and undergoes rapid metabolism, with peak metabolite concentrations reached within 30 min post-administration (Grzeczka et al. 2024). Following intestinal absorption, RES is extensively metabolized via glucuronidation and sulfation (Grzeczka et al. 2024). It has been reported to modulate CYP activity, including the induction of CYP1A2 and the inhibition of CYP3A4, CYP2D6, and CYP2C isoforms. Additionally, RES can induce the activity of phase II detoxification enzymes such as UDPGTs, GSTs, and quinone reductases (Chow et al. 2010). In breast cancer models, RES exerts multi-targeted antitumor effects by modulating key processes, including apoptosis, cell cycle regulation, autophagy, glycolysis, EMT, metastasis, migration, BCSC survival, and therapy resistance. These effects involve increased autophagy-associated LC3-II levels, the regulation of multiple signaling pathways and molecular targets, such as suppression of PI3K/Akt, Wnt/β-catenin, NF-κB, STAT3, Notch1, c-Myc, hypoxia-inducible factor-1α (HIF-1α), and MMP-9, alongside upregulation of tumor-suppressive factors, including sirtuin 1 (SIRT1), p21, E-cadherin, and pro-apoptotic proteins (Fu et al. 2014; Behroozaghdam et al. 2022). RES has been shown to induce inhibition of cyclin D, CDK4, induction of p53, activation of caspase-9 and downregulation of Bcl-2, Bcl-xL and increase of Bax (Kim et al. 2004; Pozo-Guisado et al. 2005). RES also influences drug resistance mechanisms through downregulation of P-gp and other multidrug resistance–associated proteins (Behroozaghdam et al. 2022).

### Toosendanin (TSN)

Toosendanin (TSN) is a triterpenoid with hepatotoxic effects, poor water solubility and a bioavailability of approximately 10% (Chen et al. 2025). TSN undergoes oxidative and dehydrogenative reactions in the liver to produce different metabolites (Wu et al. 2013). TSN inhibits CYP3A, enzymes involved in TSN metabolism (Li et al. 2024). In breast cancer, it induces mitochondrial apoptosis and autophagy, enhances chemosensitivity to adriamycin (ADM) via PI3K/Akt pathway inhibition, and increases responsiveness to irinotecan by suppressing irinotecan-induced autophagy, as indicated by the accumulation of LC3-II and p62 (Zhang et al. 2022c; Hu et al. 2023). Moreover, induction of caspases and decreased levels of Bcl-xL in breast cancer cells have been found after treatment with TSN (Zhang et al. 2022a). Furthermore, TSN has been shown to enhance the antitumor efficacy of paclitaxel (PTX) in TNBC cells (Li et al. 2024). In CMT-U27 cells, TSN induced apoptosis with upregulation of cytochrome C (cyt C), p53, and BAX and the downregulation of Bcl-2 (Yang et al. 2023).

### Zerumbone (ZER)

Zerumbone (ZER) is characterized by poor aqueous solubility, limited intestinal absorption, and low oral bioavailability. To address these pharmacokinetic limitations, nanostructured lipid carriers (NLCs) have been developed, offering improved delivery by bypassing first-pass metabolism (Ibáñez et al. 2023). ZER has been reported to induce phase II detoxifying enzymes, including GST and NAD(P)H:quinone oxidoreductase (NQO) (Nakamura et al. 2004). In breast cancer models, ZER enhances the cytotoxic efficacy of paclitaxel (Li et al. 2023), and suppresses pro-metastatic and pro-inflammatory mediators such as NF-κB, MMP-3, IL-8, and IL-1β, thereby inhibiting migration and invasion of TNBC cells (Girisa et al. 2019). Furthermore, ZER downregulates TGF-β1, MMP-2, MMP-9, and the proliferation marker Ki-67, while reducing Smad3 phosphorylation, collectively contributing to its antimetastatic and antiproliferative effects (Girisa et al. 2019). In CMT cells, ZER has been shown to induce apoptosis by upregulating the pro-apoptotic protein Bax, downregulating the antiapoptotic protein Bcl-2, and enhancing caspase activation (Kalantari et al. 2017; Valdivia et al. 2021).

**Fig. 2:**
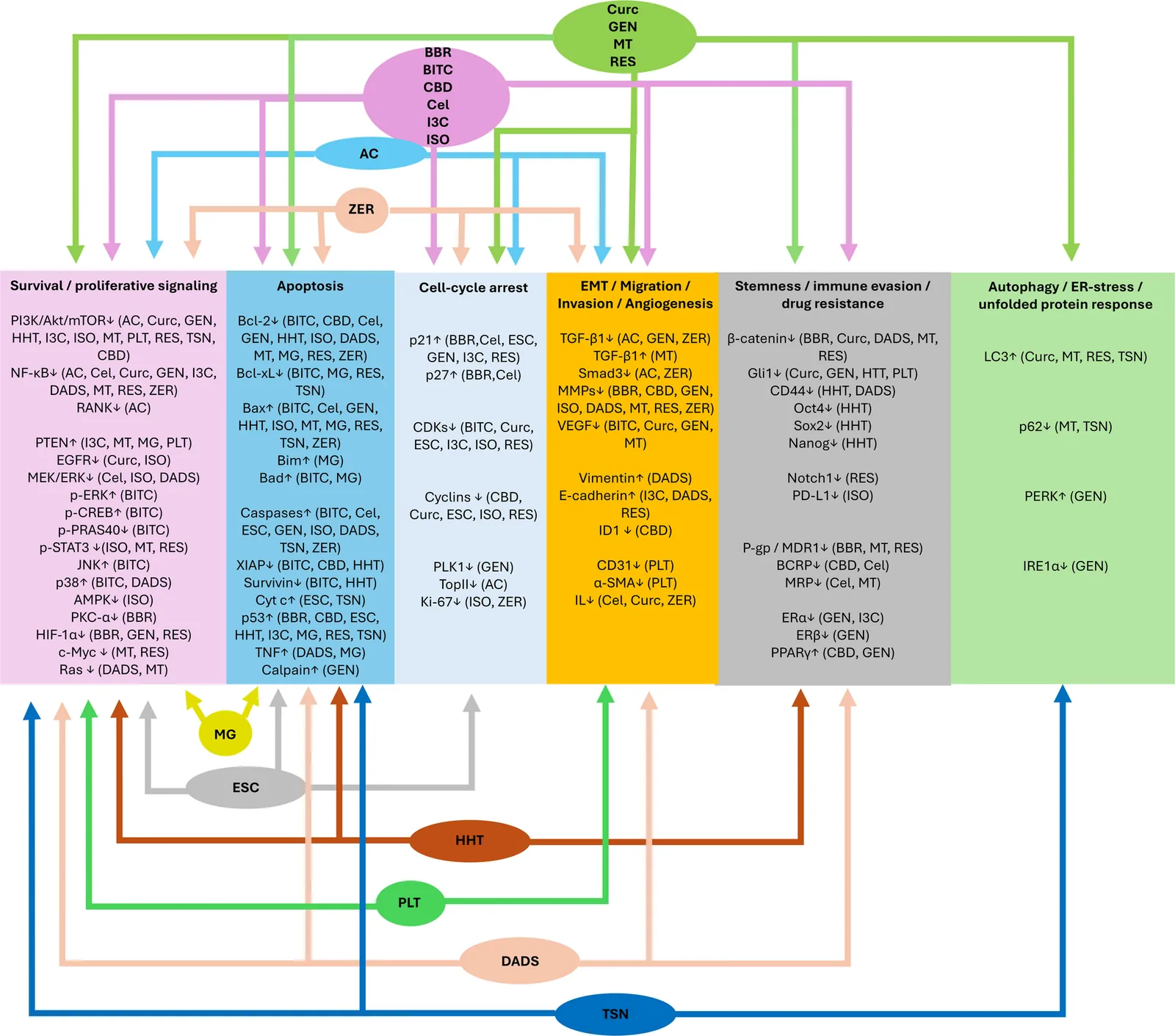
Representative overview of the main molecular and phenotypic processes reported to be modulated by phytochemicals in mammary cancer. The figure summarizes signaling pathways, and tumor-related processes affected by the phytochemicals discussed in this review. Arrows indicate reported associations of individual compounds with one or more functional categories. This scheme provides a representative mechanistic summary. Abbreviations: AC, aconitine; BBR, berberine; BITC, benzyl isothiocyanate; CBD, Cannabidiol; Cel, celastrol; Curc, curcumin; DADS, diallyl disulfide; ESC, esculetin; GEN, genistein; HHT, homoharringtonine; I3C, indole-3-carbinol; ISO, isorhamnetin; MG, methyl gallate; MT, matrine; PLT, palmatine; RES, resveratrol; TSN, toosendanin; ZER, zerumbone; α-SMA, α-smooth muscle actin; Akt, protein kinase B; AMPK, AMP-activated protein kinase; Bad, Bcl-2-associated agonist of cell death; Bax, Bcl-2-associated X protein; Bcl-2, B-cell lymphoma 2; Bcl-xL, B-cell lymphoma-extra large; BCRP, breast cancer resistance protein; CD31, cluster of differentiation 31; CD44, cluster of differentiation 44; CDKs, cyclin-dependent kinases; CREB, cAMP response element-binding protein; Cyt c, cytochrome c; E-cadherin, epithelial cadherin; EGFR, epidermal growth factor receptor; ERα, estrogen receptor α; ERβ, estrogen receptor β; ERK, extracellular signal-regulated kinase; Gli1, glioma-associated oncogene homolog 1; HIF-1α, hypoxia-inducible factor 1α; ID1, inhibitor of DNA binding 1; IL, interleukin; IRE1α, inositol-requiring enzyme 1α; JNK, c-Jun N-terminal kinase; Ki-67, marker of proliferation Ki-67; LC3, microtubule-associated protein 1 light chain 3; MDR1, multidrug resistance protein 1; MEK, mitogen-activated protein kinase kinase; MMPs, matrix metalloproteinases; mTOR, mechanistic target of rapamycin; MRP, multidrug resistance-associated protein; NF-κB, Nuclear factor kappa-light-chain-enhancer of activated B cells; Notch1, notch receptor 1; Oct4, octamer-binding transcription factor 4; p21, cyclin-dependent kinase inhibitor 1 A; p27, cyclin-dependent kinase inhibitor 1B; p38, p38 mitogen-activated protein kinase; p53, tumor protein p53; p62, sequestosome 1; P-gp, P-glycoprotein; PI3K, phosphoinositide 3-kinase; PKC-α, protein kinase C-α; PLK1, polo-like kinase 1; PPARγ, peroxisome proliferator-activated receptor gamma; PRAS40, proline-rich Akt substrate of 40 kDa; PTEN, phosphatase and tensin homolog; RANK, receptor activator of nuclear factor kappa B; Ras, rat sarcoma; Smad3/4, SMAD family member 3/4; Sox2, SRY-box transcription factor 2; STAT3, signal transducer and activator of transcription 3; TGF-β1, transforming growth factor-β1; TNF, tumor necrosis factor; TopII, topoisomerase II; VEGF, vascular endothelial growth factor; XIAP, X-linked inhibitor of apoptosis protein

Compared to crude extracts, isolated phytochemicals offer a more convenient means to elucidate specific mechanistic patterns of antitumor activity. For the reviewed mechanistic evidence was drawn from breast cancer models in general. Mechanistically, these compounds have been reported to act through partially overlapping but recurrent antitumor pathways. Aconitine, celastrol, Curc, genistein, HHT, ISO, MT, MG, PLT, RES, and TSN were linked mainly to suppression of survival signaling, particularly PI3K/Akt/mTOR and NF-κB-related pathways. BITC, BBR, celastrol, Curc, esculetin, genistein, HHT, ISO, DADS, MT, MG, RES, TSN, and ZER promoted apoptosis and/or cell-cycle arrest through regulation of Bcl-2 family members, caspases, p53/p21/p27, and cyclins/CDKs. Antimetastatic and antiangiogenic effects were reported for several compounds through modulation of TGF-β/Smad, MMPs, VEGF, and EMT-associated mediators such as snail, twist, and vimentin. Additional mechanisms included inhibition of stemness- and resistance-related signaling, including Wnt/β-catenin, Hh/Gli1, PD-L1, CD44, Oct4, Sox2, Nanog, and multiple drug-resistance transporters, while some compounds also modulated autophagy and unfolded protein response pathways (Fig. 2).

## Safety, interactions, and translational barriers

As discussed above, plant extracts and certain phytochemicals may exert multimodal biological effects (El-Saadony et al. 2025), and several have shown promising activity in vitro and in vivo canine mammary cancer models. Nevertheless, these same properties may also complicate clinical translation, as multi-target activity and modulation of metabolic enzymes can increase the risk of toxicity, variable pharmacokinetics, and drug interactions. Moreover, only one in vitro experiment was identified evaluating the use of plant-derived products on feline mammary cancer cells and no significant potency was demonstrated (Deveci Ozkan et al. 2021). This is particularly important, as the pharmacokinetic and metabolic profiles may differ substantially between dogs and cats, making direct extrapolation unsafe.

### Toxicity and safety considerations

#### Toxicity and safety of plant extracts

Among the extracts discussed in this review, several raise noteworthy safety concerns. *Calotropis procera* is known to potentially exert toxic effects in humans and animals, including mice and rabbits, with toxicity mainly attributed to cardiotoxic cardenolides that inhibit the Na^+^/K^+^-ATPase pump (Al-Zuhairi et al. 2020; Iyadurai et al. 2020). Although acute oral toxicity studies of *Dendrophthoe pentandra* extracts in mice and rats yielded relatively high LD₅₀ values (8.81–17.78 g/kg), subchronic administration at 420 mg/kg/day for 90 days in Wistar rats was associated with histopathological abnormalities in the liver, kidney, and heart (Mustarichie et al. 2016). *Euphorbia* species have also been associated with mild toxicity in cats and dogs. Despite the fact that death from ingestion have not been reported, the plant contains euphorbol esters and steroids that irritate the skin, and mucous membranes inducing dermatitis, gastritis, vomiting, diarrhea, hypersalivation, and conjunctivitis among other symptoms (Bertero et al. 2020). Similarly, *Viscum album* has been associated with poisoning incidents in dogs and cats, with reported gastrointestinal, neurological, and in severe cases fatal outcomes following ingestion of mistletoe preparations containing berries, although a systematic review concluded that therapeutic use in companion animals is generally well tolerated, with mostly scarce and self-limiting adverse effects (Campbell and Chapman 2000; Caloni et al. 2013; Biegel et al. 2022).

Some products appear to have more favorable or better-characterized safety profiles, although caution remains necessary. *Camellia sinensis-*derived products appear to be tolerated by dogs and cats under typical dietary exposure conditions, although safety depends on dose, formulation, and feeding status. In particular, epigallocatechin gallate (EGCG) showed a no-observed-adverse-effect level (NOAEL) in nonfasted dogs that was approximately tenfold higher than that observed in fasted dogs (Kapetanovic et al. 2009).

*Curcuma longa* and *Piper nigrum* extracts may be used as an additive in animal diet and its safe concentrations expressed as mg/kg feed have been established by the European Food Safety Authority (EFSA), depending on animal exposure through diet per day (EFSA Panel on Additives and Products or Substances used in Animal Feed (FEEDAP) et al. 2020, 2022a). *Punica granatum* extract has been studied as a water additive for canine dental health, and no adverse effects were reported in that study (Gawor et al. 2023). When pomegranate extract was tested in rats no adverse effects were observed up to the highest dose tested (600 mg/kg/day for 90 days) (Patel et al. 2008). Rosemary is listed by the American Society for the Prevention of Cruelty to Animals (ASPCA) as non-toxic to dogs and cats (American Society for the Prevention of Cruelty to Animals n.d.), with EFSA proposing maximum safe concentrations of 300 mg/kg complete feed for dogs and 50 mg/kg complete feed for cats (EFSA Panel on Additives and Products or Substances used in Animal Feed (FEEDAP) et al. 2022b). Plantamajoside, a major glycoside of *Plantago asiatica* showed no observed adverse effects in rats up to 2 g/kg (Park et al. 2007), while *P. ovata* has been accepted by EFSA as a sensory additive in dog and cat feed within defined limits (EFSA Panel on Additives and Products or Substances used in Animal Feed (FEEDAP) et al. 2021).

For several products, however, safety data remain limited or insufficiently characterized. *Gynura procumbens* not listed by ASPCA as toxic to dogs and cats, and one case report described daily administration to a dog with mast cell tumor as well tolerated, but broader toxicological evidence in companion animals remains sparse (Kunakornsawat and Jermnak 2024). Similarly, safety information for *Typhonium flagelliforme* in dogs and cats remains limited, despite a reported LD_50_ of 48 g/kg in rats (Murwanti et al. 2023).

#### Toxicity and safety of phytochemicals

Among the phytochemicals discussed in this review, some compounds exhibit clear and well-documented toxicity, whereas others appear to be better tolerated under specific conditions, and some remain insufficiently characterized in dogs and cats. In addition, for some phytochemicals or phytochemical-containing preparations, safety has also been assessed in a regulatory feed-related context, which should be interpreted separately from dedicated toxicological studies.

Aconitine, a highly toxic alkaloid, has exhibited an LD_50_ of 1.8 mg/kg in mice, while in humans lethality may occur from doses of approximately 1 mg (Zheng et al. 2025). Its toxicity is attributed to the persistent activation of sodium channels, causing cardiotoxic and neurotoxic effects (Chan 2009). Aconitine poisoning may also occur transdermally, especially when skin integrity is compromised (Zheng et al. 2025). Given its well-recognized toxicity, it is subject to strict regulatory restriction in some jurisdictions (Ye et al. 2013).

Homoharringtonine has been evaluated in dogs, and the available nonclinical data indicate a clear toxicity liability, including cardiovascular involvement. Following a single intravenous dose of 0.32 mg/kg HHT in 5% ethanol, Beagle dogs exhibited marked cardiac abnormalities, and all animals either died prematurely or were sacrificed moribund. In a 6-month repeat-dose dog study, early deaths occurred at higher dose levels, and major targets of toxicity included the bone marrow, cardiovascular system, and lymphoid tissues, with additional findings involving the lungs, kidneys, liver, intestinal tract, and clinical pathology parameters suggestive of renal and/or hepatic impairment (Kantarjian et al. 2001; U.S. Food and Drug Administration 2012).

Toosendanin is considered a hepatotoxic compound. Intraperitoneal administration of TSN at 5–20 mg/kg for 24 h induced hepatotoxic effects in mice, including enzyme elevation and histologic evidence of liver injury, with hepatocyte necrosis in the highest-dose group (Luo et al. 2022). Moreover, TSN has been shown to induce time- and dose-dependent liver toxicity in zebrafish treated with 10–100 μg/mL TSN (Sun et al. 2021).

Matrine toxicity has been evaluated in mice, and an LD_50_ of 157 mg/kg was reported, accompanied by degenerative changes in nerve cells within brain tissue (Wang et al. 2010). Matrine has also been associated with hepatotoxicity, neurotoxicity, and developmental toxicity (Li et al. 2021a). Moreover, teratogenicity and death have been reported in zebrafish, with EC_50_ values of 145 mg/L and 240 mg/L, respectively (Lu et al. 2014).

Other compounds appear to have a more favorable or dose-dependent safety profile, although adverse effects may still emerge depending on dose, route of administration, or duration of exposure. CBD is considered relatively safe in dogs and cats, with available pharmacokinetic and toxicity data (Hommerding 2026). In a study including 8 cats oral administration of CBD up to 80 mg/kg no clinically relevant adverse effects were reported, only transient administration-related signs, including head shaking, lip smacking, and hypersalivation, were observed immediately after dosing (Rozental et al. 2023). n dogs, CBD is generally considered to have low acute toxicity, with an intravenous LD50 exceeding 254 mg/kg and a reported NOAEL of 100 mg/kg. However, mild gastrointestinal signs and, less frequently, somnolence, lethargy, or ataxia have been reported in clinical studies, particularly depending on formulation and cannabinoid composition (Di Salvo et al. 2023).

BITC safety has been evaluated in mice and rats. When 12 μmol of BITC were orally administered daily for 46 days, no toxicity was observed (Boreddy et al. 2011). n rats, oral administration of BITC up to 50 mg/kg did not cause fetal loss; however, fetal weights were lower at the higher doses of 25 and 50 mg/kg, signs of toxicity were observed, and three deaths occurred (Adebiyi et al. 2004). In another study, BITC was administered to rats for 4 weeks at doses of 50–200 mg/kg. A decrease in body weight was observed even at 50 mg/kg, hematological changes occurred at doses of 100 mg/kg and above, and abnormalities in urine analysis and organ morphology were detected even at the lowest dose after 3 weeks (Lewerenz et al. 1992).

Celastrol has been administered in food at a dose of 0.28–0.41 mg/kg/day for 24 weeks and no adverse effects were observed. However, gastrointestinal, hepatic, and respiratory adverse effects have been observed in rodent studies when doses exceeding 7.5 mg/mL were administered (Shin et al. 2026). Moreover, in different animal models, celastrol has been linked a variety of adverse effects in cardiovascular system in zebrafish, rats and mice and reproductive toxicity in guinea pigs (Wang et al. 2023a).

Berberine has shown toxic effects in companion animals. When orally administered to cats at doses of 50 and 100 mg/kg for 10 days, intestinal hemorrhagic inflammatory lesions were observed, whereas 25 mg/kg did not produce such effects. In dogs, oral administration of 2.75 g of berberine resulted in gastrointestinal symptoms, muscular tremor, and paralysis. Moreover, intravenous administration has been associated with vasodilation and cardiac depression in dogs (Rad et al. 2017).

Genistein safety has been evaluated in dogs at both chronic (52 weeks) and subchronic (4 weeks) levels through oral administration. Doses up to 500 mg/kg/day were well tolerated, while the reproductive system was affected in the groups receiving 150 and 500 mg/kg/day in both female and male dogs; however, no systemic toxicity was detected. The NOAEL was considered to be above 500 mg/kg/day, while the NOEL was 50 mg/kg/day (McClain et al. 2005). In cats, long-term (> 1 year) oral administration of dietary genistein did not result in statistically significant differences in hepatic enzymes, bile acids, or histological parameters (Whitehouse-Tedd et al. 2012). In rats, oral administration of genistein induced changes in the reproductive organs, bone, kidneys, heart, liver, and spleen, which were considered hormonally related due to its phytoestrogenic effect. The NOAEL was considered to be 50 mg/kg/day and the NOEL 5 mg/kg/day (McClain et al. 2006).

I3C has been examined in dogs receiving oral doses of 4, 20, and 100 mg/kg/day for 13 weeks. Dogs administered 100 mg/kg/day exhibited anorexia, dehydration, weight loss, and gastrointestinal symptoms, and the high dose was reduced to 50 mg/kg/day for the final 7 weeks. Histopathological changes of the gall bladder epithelium were observed at all doses, while high doses also induced lesions in renal cortical and gastric cells, increased liver weight, and hyperbilirubinemia in females (National Toxicology Program 2008).

Resveratrol has been administered to cats in a study evaluating the effect of supplementation on lipid metabolism. Cats received either 1 or 5 mg/kg/day for 4 weeks, however, potential treatment-related adverse effects were not described in that study (Yun et al. 2025). In a subchronic toxicity study in dogs, daily oral administration of RES at doses up to 1.2 g/kg/day for 90 days revealed minimal toxicity, mainly a reduction in body weight gain, while limited histopathologic changes in the kidney and urinary bladder were considered not toxicologically significant. The NOAEL was estimated to be 600 mg/kg/day in dogs (Johnson et al. 2011).

For several other phytochemicals, species-specific safety data in dogs and cats remain limited, and the available evidence derives mainly from rodents or other experimental models. For esculetin, species-specific toxicological data in dogs and cats appear to be very limited. The available evidence comes mainly from rodent. In mice, no mutagenicity or bone marrow cytotoxicity was reported after oral administration of 25, 50, and 500 mg/kg (Marques et al. 2015). Moreover, oral administration showed an LD_50_ > 2 g/kg, whereas intraperitoneal administration exhibited an LD_50_ of 1.45 g/kg (Tubaro et al. 1988).

Palmatine toxicity has been evaluated in rodents. Acute toxicity studies have estimated LD_50_ of approximately 1.5 g/kg when PLT was administered orally to mice. In a subchronic toxicity test, 156 mg/kg of PLT was administered daily to rats through the diet for 90 days. Changes in body and organ weights, hematological parameters, and some biochemical parameters, including ALT, AST, and γ-GT, were observed. However, no gross or histological differences were identified, and no deaths were reported (Yi et al. 2013).

Organosulfur compounds are found abundantly in *Allium* spp., and some of them are oxidizing compounds that have been associated with the oxidative hemolysis observed in garlic and onion poisoning in dogs and cats. Specifically, toxicosis may occur in cats after onion consumption of at least 5 g/kg, while in dogs ingestion of 15–30 g/kg may result in toxic events (Salgado et al. 2011). In a 12-week study, up to 90 mg/kg of aged garlic extract was orally administered to dogs and no adverse effects were observed, while antioxidant enzymes were upregulated in whole blood samples (Yamato et al. 2018). In dogs, however, toxic effects of garlic extract have been reported at doses higher than 1.25 mL/kg of garlic extract, or 5 g/kg of fresh garlic daily for seven days (Beleć et al. 2025). In mice administered diallyl sulfide at doses above 1.6 g/kg, death occurred, with the lungs, liver, and reproductive organs being affected at higher doses (Dutta et al. 2021).

Although species-specific safety data remain limited, one mouse study reported no overt toxicity after a single intraperitoneal dose of zerumbone at 500 mg/kg or after repeated intraperitoneal administration of 5–50 mg/kg over 28 days, with no significant treatment-related changes in body weight, hematology, serum biochemistry, organ weights, or histopathology(Jin et al. 2013). In a separate genotoxicity study, ZER administered intraperitoneally to rats at 250–1,000 mg/kg increased the number of micronuclei in polychromatic erythrocytes at the highest dose, while the maximum tolerable dose was estimated at 1,000 mg/kg and the lethal dose at 2,000 mg/kg (Al-Zubairi et al. 2010).

In addition to experimental toxicology studies, some phytochemicals or phytochemical-containing products have also been evaluated in a regulatory or feed-related safety context. As mentioned above, *Curcuma longa* has been established as a potential feed additive at certain concentrations. Nevertheless, intravenous administration of liposomal curcumin (Lipocurc) in dogs has been associated with hemolysis, allergic reactions, increased AST, and vomiting (Helson et al. 2012; Withers et al. 2018).

Although ISO safety has not been fully evaluated, EFSA has deemed safe maximum concentration of *Ginkgo biloba* extract which contain approximately 2–3% isorhamnetin glycosides, at 3.3 mg/kg complete feed for dogs and 2.8 mg/kg complete feed for cats ((FEEDAP) et al. 2024a). Outside dogs and cats, isorhamnetin has been evaluated mainly in rodent efficacy models rather than in dedicated toxicology studies. In these experiments, rats treated with isorhamnetin at pharmacologically relevant doses generally showed protective or therapeutic effects, when administered intraperitoneally at 5 mg/kg, 25–100 mg/kg intragastrically (Sun et al. 2013; Dong et al. 2022).

Propyl gallate appears to have a better characterized safety profile than methyl gallate, as regulatory evaluations have identified subchronic rat toxicology data sufficient to derive safe concentrations for use in complete feed for dogs and cats. Dietary exposure studies indicated that 71 mg/kg complete feed is safe for both cats and dogs ((FEEDAP) et al. 2024b). In an in vivo dog study, 200 mg/kg of propyl gallate administered orally in enteric-coated tablets daily for 27 days induced nephrotoxicity; however, nephrotoxicity was not observed when the compound was administered in an immediate-release capsule (Mou et al. 2025).

Available safety data remain heterogeneous and are often derived from different species, exposure routes, and clinical contexts, underscoring the need for species-specific toxicological and pharmacokinetic evaluation before veterinary clinical translation.

### Herb-drug interactions and pharmacokinetic considerations

Plant-derived products may interact with conventional drugs either directly or indirectly, resulting in synergistic, antagonistic, or pharmacokinetic interactions (Posadzki et al. 2013). Natural products may affect drug absorption, distribution, metabolism, and excretion through multiple mechanisms. These include modulation of membrane transport systems such as P-gp, competitive binding to plasma proteins such as albumin, inhibition or induction of drug-metabolizing enzymes, and effects on transporter activity or urinary excretion (Chaachouay 2025). Such interactions may be clinically insignificant when they do not affect treatment efficacy or patient safety. However, in some cases, they may lead to life-threatening situations or even death (Gouws and Hamman 2020). These interactions are particularly important when cancer chemotherapeutic drugs with a narrow therapeutic index are used. Small changes in the concentrations of drugs with a narrow therapeutic window may result in therapeutic failure or toxic events. Plant-derived products may also contribute to polypharmacy and thereby increase the risk of interactions (Gouws and Hamman 2020). One comprehensive resource for plant product interactions with drugs is the *Natural Medicines Comprehensive Database* (Hsu 2002; Gouws and Hamman 2020). Among the products reviewed here, several plant extracts and phytochemicals with greater translational relevance warrant caution in combination settings.

Some of the more relevant plant extracts discussed in this review have documented or plausible interaction potential through modulation of metabolic enzymes or transporters. *Camellia sinensis* is known to affect the bioavailability of several drugs by modulating organic anion transporting polypeptides (OATP), P-gp, and UDPGT. Thus, it may interact with several drugs, although no change in tamoxifen area under the curve (AUC) was reported in one study (Kyriacou et al. 2025). Green tea has also demonstrated a synergistic effect against breast cancer cells and in a mouse xenograft model (Sartippour et al. 2006), while its consumption increased plasma concentrations of 5-fluorouracil (5-FU) in rats (Qiao et al. 2011). *Curcuma longa* extracts and curcumin have been shown to modulate various metabolic enzymes, including CYP enzymes, P-gp, MRP1, UDPGT, sulfotransferases, GST, and OATP. Rodent studies have demonstrated pharmacokinetic interactions of curcumin with antineoplastic agents such as paclitaxel, docetaxel, etoposide, tamoxifen, everolimus, and phospho-sulindac (Bahramsoltani et al. 2017). At the same time, curcumin has exhibited protective effects against doxorubicin-induced cardiotoxicity in rats (Akca et al. 2025), while it has also been shown to reverse doxorubicin resistance in breast cancer cells (Wen et al. 2019).

The potential interactions of *Euphorbia royleana* have not yet been fully described, however, in silico models have predicted activity on different metabolizing enzymes (Kgosiemang et al. 2025), and an in vitro assessment of isolated diterpenoids derived from the plant demonstrated enhancement of doxorubicin cytotoxicity, with possible inhibition of P-gp (Shaker et al. 2020). Some interactions have also been described for *Rosmarinus officinalis* extracts and their constituents. Specifically, rosemary extract has been shown to induce inhibition of P-gp activity and increased accumulation of doxorubicin and vinblastine in drug-resistant breast cancer cells, but not in wild-type cells (Ciolino et al. 1999). In addition, both in vitro and in vivo modulation of CYP enzymes has been reported for rosemary extract and its major active constituent, rosmarinic acid (Cho and Yoon 2015). Piperine, a major constituent of *Piper nigrum*, affects various enzymes comprehensively described by Han (2011). Among its effects, it exhibits concentration-dependent effects on CYP enzymes and inhibits UDPGT and hepatic aryl hydrocarbon hydroxylase activity in rats. Dietary administration of *P. nigrum* has also been associated with increased GST activity in mice, and there are further indications of P-gp modulation by piperine (Han 2011). *Punica granatum* has been shown to modulate CYP enzymes both in vitro and in vivo in rodents by inhibiting CYP1A2, CYP2C9, and CYP3A, however, in human clinical trials, consumption of pomegranate juice did not significantly affect CYP3A activity (Razzaghi et al. 2026). In addition, one study indicated that pomegranate juice exerted protective activity against doxorubicin-induced cardiotoxicity in rats (Hassanpour Fard et al. 2011).

Direct herb–drug interaction data for *Plantago asiatica* appear limited, although some evidence suggests possible relevance. Plantamajoside has been reported to increase plasma concentrations of nifedipine, reduce its clearance, and inhibit CYP3A (Huang et al. 2026). Moreover, plantainoside D inhibited CYP1A2, CYP2D6, and CYP3A in vitro (Zhou et al. 2022).

Phytochemicals discussed in this review may also have interaction relevance through effects on drug-metabolizing enzymes or transporters. Aconitine has been shown to induce P-gp expression both in vitro and in vivo in mice reducing susceptibility to acute toxic injury. These findings suggest that co-administration of drugs that are P-gp substrates or modulators may have interaction potential (Wu et al. 2016). Celastrol absorption may be inhibited by diclofenac administration in rats, as diclofenac induces the activity of P-gp (Wang et al. 2018a). Celastrol has been shown to inhibit CYP3A4, CYP2C19, CYP2D6, CYP1A2, and CYP2E1 in vitro (Jin et al. 2015) and to inhibit P-gp activity in doxorubicin-resistant cells (Moreira et al. 2018). Although BBR is not among the most promising phytochemicals based on in vitro results, it represents a useful example of drug-phytochemical interactions. BBR is metabolized primarily by CYP2D6 and secondarily by CYP1A2, thus, inhibitors of these enzymes such as quinidine may inhibit its metabolism. Moreover, urinary excretion of BBR is mediated by organic cation transporter 2 (OCT2) and multidrug and toxin extrusion protein 1 (MATE1). OCT2 inhibitors such as corticosterone and MATE1 suppressors such as pyrimethamine decrease BBR excretion. BBR is a P-gp suppressor and may enhance the bioavailability of digoxin, as demonstrated in rats (Khoshandam et al. 2022). Cannabinoids, including CBD are metabolized by CYP and UDPGT enzymes and modulate several isoforms, including CYP2C9, CYP3A4, CYP2D6, and CYP2C19, while CBD also inhibits different UDPGT isoforms and carboxylesterase 1 (CES1). Accordingly, pharmacokinetic interactions have been reported with NSAIDs, immunosuppressants, and sedatives (Czigle et al. 2023).

I3C exerts various effects on hepatic drug-metabolizing enzymes in mice, including induction of CYP1A-associated activity, increased estradiol 2-hydroxylase and testosterone 6α-hydroxylase activities, and enhanced NADPH-cytochrome P450 reductase activity, while its acid condensation products inhibited testosterone 6β-hydroxylase in vitro (Baldwin and LeBlanc 1992). Dietary administration to rats increases CYP2B1/2, CYP3A1/2 hepatic levels, and inhibits both the levels and the activity of flavin-containing monooxygenase (FMO) in the liver and intestine (Larsen-Su and Williams 1996). In a phase I study in women at high risk for breast cancer I3C increased lymphocyte GST activity and markedly induced CYP1A2 (Reed et al. 2005). In a study in female rats, TSN significantly altered the pharmacokinetics of tamoxifen, increasing its maximum plasma concentration and half-life while decreasing its clearance. In vitro studies suggested that these effects may be related to CYP2D6 inhibition and reduced metabolic turnover of tamoxifen (He et al. 2025).

Palmatine, while being a P-gp substrate, has not shown inhibitory effects on P-gp (Zhang et al. 2011). However, it has been associated with in vitro inhibition of CYP1A1, CYP1B1, CYP2D6, and CYP3A4 while also activation of AhR, CYP2C9, CYP2C19 and induction of CYP1A1 expression (Vrba et al. 2015; Long et al. 2019; Tarabasz and Kukula-Koch 2020). Dietary administration of DADS to rats has been shown to enhance intestinal epoxide hydrolase, CYP2B1/2 protein levels, aryl hydrocarbon hydroxylase, UDPGT, GST, and O-dealkylase activities, while decreasing nitrosodimethylamine demethylase activity. In the liver, CYP1A1/2 protein levels were increased along with O-dealkylase activities and UDPGT, whereas CYP2E1 levels were decreased (Haber et al. 1995). Zerumbone inhibited UDPGT activity in human and rat liver microsome in vitro, however different *IC*_50_ values were observed, another indication warranting caution extrapolating results in between species (Abdullah and Ismail 2018). ZER has also been reported to induce phase II detoxification enzymes, including GST, however CYP1A1 was not affected (Nakamura et al. 2004), although direct evidence for clinically relevant herb–drug interactions remains limited. Isorhamnetin has exhibited inhibitory effects on CYP1A2, CYP2A6 (Bojić et al. 2019). In addition, its absorption and transport appear to depend on efflux transporters. When isorhamnetin was co-administered with quercetin in rats, both compounds showed increased maximal plasma concentration and area under the curve (AUC) (Lan et al. 2008).

Although direct veterinary interaction studies on dogs and cats remain limited, the available evidence suggests that plant-derived agents may alter the disposition, efficacy, or toxicity of co-administered drugs through modulation of metabolic enzymes and transporters. These considerations may be particularly relevant when plant-derived agents are combined with conventional drugs used in veterinary mammary cancer management.

### Standardization, quality control, and formulation barriers

As already mentioned, plant-derived extracts demonstrate variability depending on multiple factors. Some of these factors can be standardized in the laboratory, such as extraction methods and conditions, whereas others include plant age, growing and environmental conditions, soil quality and consistency, climate, and harvest time (Patnala and Kanfer 2021). In addition, different extracts of the same plant may show variation in biological activity because they contain numerous compounds whose concentrations may differ substantially (Patnala and Kanfer 2021). To add to this complexity, in some products the active constituents remain poorly characterized (Kaundal and Kumar 2025). In several countries, plant-derived medicines are not regulated under the same principles of quality, safety, and efficacy as conventional pharmaceuticals. For example, the European Medicines Agency (EMA) approaches herbal medicinal products as phytomedicines, whereas the U.S. Food and Drug Administration (FDA) often regulates them as dietary supplements (Kaundal and Kumar 2025).

Complete identification of the identity, purity, and authenticity of the plant, as well as the plant part used, together with standardization of growing conditions, storage, processing, extraction techniques, and analytical methods to quantify active ingredients, may raise quality standards and minimize batch-to-batch variability (Wang et al. 2023b). In addition, contamination control, including the removal of dirt and debris and the use of cleaning, disinfection, or sterilization techniques when necessary, may further improve safety and product quality (Wang et al. 2023b). Identification and isolation of specific active phytochemicals may also reduce variability while strengthening standardization and improving consistency of biological activity.

A further barrier to clinical translation is the poor bioavailability of some phytochemicals, which may restrict their use even when strong mechanistic activity is observed in preclinical models. To overcome limitations in drug delivery, distribution, and systemic exposure, different formulation strategies may be employed. A characteristic example is paclitaxel, whose oral administration results in poor bioavailability because of limited absorption and extensive first-pass metabolism, thereby necessitating intravenous formulation (Kim et al. 2025). Other possible approaches include chemical structure modification, prodrug development, and nanotechnological strategies such as liposomes, phytosomes, and protein-based nanoformulations, all of which may improve bioavailability and delivery (Singh et al. 2024; Kim et al. 2025).

## Molecular and microenvironmental features relevant to phytochemical targeting in canine and feline mammary tumors

### Molecular classification

Canine mammary carcinomas can be classified into molecular subtypes similarly to HBC, based on the expression of ER, progesterone receptor (PR), and human epidermal growth factor receptor 2 (HER2). Thus, luminal A-like (ER + and/or PR +, HER2 −), luminal B-like (ER + and/or PR +, HER2 +), HER2-overexpressing (ER −, PR −, HER2 +), and triple-negative (ER −, PR −, HER2 −) phenotypes, or modifications of these categories, may be applied (Varallo et al. 2019; Nosalova et al. 2024; Razavirad et al. 2024; Ferreira et al. 2025). However, HER2 does not yet have an established clinical role in canine mammary tumors (Muscatello et al. 2022). Accordingly, additional biomarkers have been investigated for their diagnostic and prognostic relevance. Positive CK19 expression has been associated with ER expression, whereas reduced CK19 expression has been linked to a more aggressive phenotype and may be associated with tumor progression (Gama et al. 2010). Mucin-1 (MUC-1) expression has been associated with higher tumor grades, invasion, and metastatic processes in CMTs (Nosalova et al. 2024). Increased Ki-67 expression has been associated with malignancy, metastasis, and poorer clinical outcome, including shorter disease-free and overall survival (Zuccari et al. 2004). In canine malignant mammary tumors, vimentin overexpression has been linked to a more aggressive phenotype, including higher grade, increased proliferation, angiogenesis, and vascular invasion (Rismanchi et al. 2014).

For feline mammary carcinoma (FMC), both a five- and a six-subtype molecular classification system, more closely related to those used in human breast cancer, have been proposed. These systems are modifications of the basic classification based on ER, PR, and HER2 expression, and include luminal A (ER + and/or PR +, HER2 −, low Ki-67), luminal B/HER2 + (ER + and/or PR +, HER2 +), HER2-positive (ER −, PR −, HER2 +), and triple-negative tumors, which may be further classified as basal-like (CK5/6, CK14, and CK17 positive) or normal-like (Wiese et al. 2013; Brunetti et al. 2013). More recently, the six-subtype system has proven useful with the addition of luminal B/HER2 − (ER + and/or PR +, HER2 −, high Ki-67) (Soares et al. 2016a). The Ki-67 index has been proposed as an indicator of disease progression risk (Soares et al. 2016b). In FMC, ER − and PR − status has also been associated with increased angiogenesis, vascularity, and VEGF expression, further supporting the link between hormone receptor loss and aggressive tumor behavior (Soares et al. 2022).

### Tumor microenvironment and immune checkpoints

Beyond molecular classification, the tumor microenvironment (TME) plays an important role in mammary carcinoma progression, invasion, angiogenesis and response to treatment. TME is composed of different types of cells including tumor cells, stromal fibroblasts, adipocytes, epithelial, endothelial and immune cells and components of the extracellular matrix (ECM). Matrix accumulation and stiffening are important processes in tumor growth and progression (Gkretsi and Stylianopoulos 2018). Similar TME-related processes described in human breast cancer include increased expression of fibronectin, periostin, tenascin-C, collagen, and vitronectin, as well as changes involving mediators such as VEGF, COX-2/prostaglandin E2 (PGE2), and immune checkpoint molecules including PD-1, PD-L1, PD-L2, cytotoxic T-lymphocyte associated protein 4 (CTLA-4), V-domain Ig suppressor of T cell activation (VISTA), and T-cell immunoglobulin and mucin-domain containing-3 (TIM-3) (Saleh et al. 2019; Curran and Ponik 2020; Zong et al. 2020; Ghahremani Dehbokri et al. 2023). Immune checkpoints play a central role in regulating inflammation and antitumor immune responses. Consequently, immune checkpoint inhibitors are being investigated as therapeutic approaches to enhance immune recognition and elimination of cancer cells. For a detailed review of immune checkpoint function and therapeutic potential in breast cancer and FMC, readers are referred to Vilela et al. (2024).

ECM remodeling also appears to contribute to CMC progression, as both tubular and solid carcinomas were positive for collagen I, collagen III, and fibronectin, while solid carcinomas additionally showed high expression of VEGF, PCNA, CK-18, and vimentin (Borghesi et al. 2021). In CMTs increased PD-L1 and CTLA-4 expression has been associated with higher grade, lymph node metastasis, and tumors that later developed metastases (Monteiro et al. 2025b). Moreover, VISTA has been reported in neoplastic and inflammatory infiltrative cells. Increased VISTA expression in tumor cells was associated with higher histological grade, but no significant association with molecular subtype was determined (Yamac et al. 2025). In CMTs, COX-2 expression has been associated with histologic features of malignancy and aggressiveness, and several studies support its role as a negative prognostic marker, although variation among histologic types warrants caution. COX-2 inhibitors are already incorporated into therapeutic protocols for female dogs. In FMTs, COX-2 is frequently expressed and linked to malignant behavior, with some studies indicating its prognostic value, although the clinical use of COX-2 inhibitors in queens has not yet demonstrated a clear survival benefit (Millanta et al. 2006; Gregório et al. 2021; Guimarães et al. 2024).

In FMC, tumor-infiltrating lymphocytes are present both intratumorally and in the stroma, supporting the contribution of the immune microenvironment to tumor behavior. HIF-1α overexpression has also been reported, although its precise role in VEGF regulation and clinicopathological behavior remains incompletely defined. While VEGF overexpression has been linked to several clinicopathological features, its prognostic significance remains inconsistent across studies. In addition, poorly defined tumor–stroma boundaries, increased collagen density, and long, thick, straight collagen fibers have been associated with poorer outcomes (Rodrigues-Jesus et al. 2025). COX-2 overexpression and α-SMA-expressing CAFs have also been associated with poorer prognosis in FMC (Guimarães et al. 2024). Several immune checkpoints have been studied in FMC. Increased serum CTLA-4 levels have been associated with FMC and with some clinicopathological features, such as small tumor size and absence of tumor necrosis, and serum CTLA-4 was also correlated with serum TNF-α and IL-6 levels (Urbano et al. 2020). Another study revealed higher serum VISTA levels in HER2-positive and triple-negative tumors, while VISTA levels correlated with other immune checkpoints, including PD-1/PD-L1, CTLA-4, LAG-3, IL-6, and TNF-α (Gameiro et al. 2021a). In a study including 48 cats, TIM-3 expression in stromal tumor infiltrating lymphocytes (TILs) and cancer cells was linked to more aggressive characteristics, whereas TIM-3 expression in total or intratumoral TILs was associated with more benign features (Valente et al. 2023). In a study including serum samples from 53 queens with FMC and 15 healthy queens, serum PD-1 and PD-L1 levels were found to be significantly higher in cats with HER2-positive and triple-negative molecular subtypes and were positively correlated with serum CTLA-4 and TNF-α levels. The same study also reported higher PD-L1 expression in cancer cells and increased percentage of PD-L1 positive TILs in HER2-positive tumors compared with triple-negative normal-like tumors (Nascimento et al. 2020). In another series of 48 rare FMCs, immunohistochemical assessment of PD-1, PD-L1, and PD-L2 showed that higher PD-1 expression in tumor cells was associated with a less aggressive phenotype, while PD-L1 expression in intratumoral TILs was linked to skin ulceration and PD-L2 expression in tumor cells to the absence of ulceration (Franco et al. 2025). Serum PD-L2 has also been evaluated in FMC, with serum PD-L2 levels found to be significantly increased in cats with mammary carcinoma compared with healthy cats, particularly in HER2-positive and triple-negative subtypes. Serum PD-L2 levels also differed between luminal A and luminal B tumors and were positively correlated with several immune and angiogenic mediators, including serum CTLA-4, TNF-α, VEGF-A, VEGFR-1, VEGFR-2, and LAG-3. Moreover, serum PD-L2 was associated with PR status, HER2 status, and Ki-67 index (João et al. 2026).

These findings suggest that the canine and feline mammary tumor microenvironment is characterized by relevant angiogenic, stromal, inflammatory, and immune-checkpoint signals, all of which may represent potential targets for future plant-derived adjuvant strategies.

### Comparative relevance for plant-derived adjuvant research

Although an exhaustive comparison of canine and feline mammary tumors is beyond the scope of this review, a concise overview is useful for interpreting the translational relevance of plant-derived compounds. Dogs and cats both develop spontaneous mammary tumors that share biologically relevant features with human breast cancer, including receptor-based subtype classification, proliferative and EMT-related markers, angiogenic signaling, and an increasingly recognized contribution of the tumor microenvironment. At the same time, important differences remain. FMCs are predominantly malignant glandular epithelial tumors and are often considered more aggressive, whereas canine mammary tumors encompass a broader histological spectrum, including complex and mixed types (Cannon 2015; Adega et al. 2016; Kwon et al. 2023).

In both cats and dogs, luminal B HER2 − and triple-negative mammary carcinomas appear to be among the most frequent immunophenotypes. In feline mammary tumors, ER-/PR- profiles are particularly common, and many cases fall within the triple-negative category, which is generally associated with poorer prognosis and fewer targeted treatment options. (Frénel and Nguyen 2023; Vazquez et al. 2023).

Comparative analysis of EMT-related markers in human breast cancer, CMT, and FMT has also revealed species-specific differences. Vimentin expression was elevated in triple-negative HBC and FMTs, whereas Ki-67 was higher in FMTs and CD44 in CMTs, supporting biologically distinct but partially overlapping EMT-related profiles (Sammarco et al. 2023b). Inflammatory mammary carcinoma, a rare but highly aggressive and metastatic form, has been described in both species, more commonly in dogs and only occasionally in cats, and remains associated with a guarded prognosis (Sorenmo et al. 2019; Pîrvu et al. 2024).

Plant-derived products have mainly been proposed to affect proliferation, EMT, angiogenesis, inflammation, chemoresistance, and immune evasion. As most of the products discussed in this review have been tested in canine models, and only one study testing curcumin on FMC cells was identified, this comparative context may help identify which phytochemicals are more likely to be relevant across species and may also support the rational extension of canine-derived findings toward future research in FMC.

## Current therapies and challenges

Current therapeutic strategies for canine and feline mammary tumors primarily include surgical treatment, systemic therapies, and radiotherapy, while immunotherapeutic approaches remain under investigation. Surgical excision may range in extent from removal of the individual nodule (lumpectomy), excision of the affected mammary gland, to removal of a regional group of glands, a unilateral chain, or even bilateral chains, depending on the tumor characteristics and clinical stage.

### Surgical excision

The American College of Veterinary Surgeons recommends surgery as the treatment of choice for mammary tumors in dogs and cats, with the exception of IMCs. Surgical management tends to be more aggressive in cats, where the majority of tumors are malignant, and more conservative in dogs, where benign tumors are relatively more common (American College of Veterinary Surgeons n.d.). The primary goal of surgical excision is either to remove the tumor with histologically clean margins or to reduce the risk of new tumor development by excising adjacent normal mammary glands (Sorenmo et al. 2019). Therefore, the extent of surgery is determined by factors such as tumor size, location, lymphatic drainage, and evidence of metastasis (Vazquez et al. 2023).

Clinical outcomes vary with surgical technique. In one study, when histologically uncharacterized tumors were excised regionally, more than half of the patients relapsed, with new tumor formation in the ipsilateral mammary tissue (Stratmann et al. 2008). After lumpectomy of malignant mammary tumors, the incidence of a second tumor exceeded 70% (Stratmann et al. 2008; Sorenmo et al. 2019). While chain mastectomy reduces ipsilateral recurrence, it does not prevent contralateral tumor development, and wider excision is associated with greater surgical stress, pain, longer operative times, and higher rates of postoperative complications (Horta et al. 2015). Nevertheless, in a cohort study of 95 dogs, bilateral excision was associated with increased survival probability, and radical mastectomy yielded better clinical outcomes (Kim et al. 2024).

The choice of surgical technique is selected based on clinical parameters such as stage, tumor size and condition (e.g., ulceration, inflammation), and tumor multiplicity (Cassali et al. 2020, 2024). In dogs, lumpectomy may be considered for small (< 1 cm), firm, and non-fixed benign nodules (Nosalova et al. 2024), whereas in cats, radical mastectomy, either unilateral or bilateral, is generally recommended regardless of tumor size or location (Cassali et al. 2020). In dogs with distant metastases, surgical excision is advised only for palliative purposes (Cassali et al. 2020).

The prognosis is strongly influenced by tumor size, clinical stage, histopathological grade, and other prognostic factors. Importantly, surgical excision alone rarely ensures complete disease control, particularly in more aggressive tumor types (Morris 2013; Cassali et al. 2020). Given these limitations in preventing recurrence and controlling metastasis, adjunctive therapies, including chemotherapy and integrative approaches, have been explored.

### Chemotherapy

Chemotherapy is most often indicated in dogs with a high risk of metastasis or confirmed metastatic disease and may be combined with non-steroidal anti-inflammatory drugs (NSAIDs) to increase treatment efficacy. In dogs, the agents most frequently used include doxorubicin, gemcitabine, carboplatin, and 5-fluorouracil (Sorenmo et al. 2019; Cassali et al. 2020). Because COX-2 is highly expressed in aggressive mammary tumors, COX-2 inhibitors are also considered as adjunctive therapy (Cassali et al. 2020).

In cats, adjuvant chemotherapy is recommended for cases with large tumors (> 3 cm), aggressive histological subtypes, or evidence of metastasis. Doxorubicin may be administered alone or in combination with cyclophosphamide or carboplatin. Carboplatin may also be used as a single agent and can be a choice for rescue therapy in cases of recurrence or metastasis. Mitoxantrone is another option, either as monotherapy or in combination with cyclophosphamide. COX-2 inhibitors may also be added to the treatment plan (Sorenmo et al. 2019; Cassali et al. 2020). Despite these options, no single chemotherapeutic protocol has been shown to be clearly superior, and overall evidence regarding efficacy in treating malignant mammary tumors remains inconclusive (Petrucci et al. 2021b; Valdivia et al. 2021; Cassali et al. 2024).

An alternative strategy is metronomic chemotherapy (MC), which involves the continuous administration of low-dose oral drugs, most often cyclophosphamide or chlorambucil but also lomustine, temozolomide, and etoposide (Petrucci et al. 2024). MC has been associated with longer survival times in dogs with mammary tumors and distant metastasis, when compared to surgery alone or surgery combined with conventional chemotherapy. Moreover, dogs treated with surgery, chemotherapy and thalidomide demonstrated even greater survival benefits (de Campos et al. 2018). MC combined with carboplatin has also improved survival outcomes compared to carboplatin alone (Machado et al. 2022). In dogs with IMC, MC achieved longer overall survival times compared with anti-inflammatory treatment alone (Alonso-Miguel et al. 2022). In contrast, in cats, MC has not been shown to provide significant benefit over surgery and adjuvant chemotherapy in terms of disease free interval and overall survival (Petrucci et al. 2021a, b, 2024).

A major challenge to chemotherapy is drug resistance, which may be intrinsic, failure to achieve remission, or acquired, commonly leading to relapse. Resistance mechanisms include the upregulation of efflux transporters such as MDR1 and MRP1, reduced drug influx due to decreased transporter activity, and intracellular drug inactivation through metabolic enzymes such as CYP, GST, and UDPGTs (Klopfleisch et al. 2016; Cravo et al. 2025). Furthermore, genetic and epigenetic alterations can modify apoptosis and DNA repair pathways, alter drug targets, or activate alternative survival cascades, thereby desensitizing tumor cells to therapy (Klopfleisch et al. 2016).

Resistance is further promoted by the presence of CSCs within mammary tumors. In a tumor, heterogeneous clones of CSCs may be found (Zhou et al. 2021). CSCs carry mutated genes and markers related to stemness that are crucial for the maintenance and resistance of tumors (Zhou et al. 2021). CSCs can self-renew, proliferate, differentiate, and remain dormant, enabling them to evade chemotherapy and radiotherapy, which primarily target proliferating cells. These cells play a central role in angiogenesis, immunity modulation, invasion, metastasis and resistance to conventional therapy (Li et al. 2021b). Consequently, targeting CSCs and overcoming chemoresistance remain major therapeutic challenges. Natural products and phytochemicals have recently been proposed as potential adjuvants to chemotherapy, as they may modulate mechanisms of resistance and affect the efficacy of conventional therapies, including chemotherapy, radiotherapy and immunotherapy (Pang et al. 2011; de Oliveira Júnior et al. 2018; Nisar et al. 2022; Roy et al. 2022; Chen et al. 2024).

### Other therapies

Radiation therapy is a local treatment modality, that uses high-energy rays to kill or inhibit the growth of cancer cells. The duration and times of treatment depend on a variety of factors, such as the tumor, staff, equipment, and technique (Karaca and Kırlı Bölükbaş 2025). Radiation is used as an adjuvant or as a palliative treatment in cases of inflammatory or metastatic carcinoma or partially resected tumors (Sakaguchi et al. 2000; Nosalova et al. 2024). In a study with a small sample of 18 dogs enrolled in total, radiation therapy seemed to increase time to progression and survival time, while being well tolerated (Rossi et al. 2018).

Immunotherapy aims to stimulate the host immune system to eliminate tumor cells and establish a sustained antitumor response, thereby reducing local invasion and metastatic spread. One promising approach is immunogene therapy, which uses genetic engineering to deliver genes encoding tumor-associated antigens, cytokines, or co-stimulatory molecules (Glikin and Finocchiaro 2025). Several experimental immunogene therapies have shown encouraging results in the treatment of CMC. For example, p62 DNA plasmid vaccination has been associated with reductions in tumor size and histological malignancy scores in treated dogs (Gabai et al. 2014; Venanzi et al. 2019). In another study, a gene therapy approach combining surgery with lipoplexes carrying canine interferon-β (cIFN-β) and HSV-thymidine kinase/ganciclovir (HSV-tk/GCV) achieved prolonged survival with good quality of life, with only minimal recurrence reported among dogs without initial metastasis (Finocchiaro et al. 2018). While additional immunotherapeutic strategies, including monoclonal antibodies and cell-based therapies, are under investigation, several challenges limit their clinical application. These include high treatment costs, restricted accessibility in veterinary practice, the potential for tumor resistance or immune evasion, and the emergence of mutations in therapeutic targets that may reduce treatment efficacy (Gameiro et al. 2021b; Cockey and Leifer 2023).

Hormonal therapy has been explored in canine and feline mammary tumors, but its clinical application remains limited due to poor efficacy and adverse effects, with most approaches still being experimental. In contrast, in benign conditions such as fibroadenomatous hyperplasia, hormonal therapy may be used, but ovariohysterectomy is considered the treatment of choice and generally achieves successful outcomes (Giménez et al. 2010; Valdivia et al. 2021).

## Limitations

The feline-specific studies remain scarce with only one isolated in vitro study identified, limiting species-balanced interpretation, while multiple plant-derived products have been evaluated in CMT models. Most included studies are in vitro or xenograft-based, with limited veterinary clinical evidence. Heterogeneity across studies in terms of experimental models and outcomes further restricts direct comparison. Many safety, pharmacokinetic and interaction data are derived from non-mammary models or from other species. These limitations represent a research gap that is especially important as species-specific metabolism, pharmacokinetics, and tumor biology may prevent direct extrapolation from other species to dogs and especially to cats that exhibit limited hepatic glucuronidation capacity.

## Future directions

Future research should prioritize species-specific preclinical evaluation, particularly in FMC, where direct evidence remains extremely limited. Studies focusing on pharmacokinetics, toxicology, and herb–drug interactions in dogs and cats are needed before clinical translation, especially for compounds with poor bioavailability or enzyme-modulating properties. Standardized botanical preparations and optimized formulations should also be developed to improve reproducibility and systemic exposure. In addition, future studies should assess combinations with conventional therapies and better align treatment strategies with molecular subtype, EMT status, and tumor microenvironmental features.

Several plant extracts and phytochemicals have been reported to modulate biologically relevant processes, including proliferation, apoptosis, angiogenesis, EMT, inflammation, and chemoresistance in preclinical models. Most of this evidence remains preclinical and is derived mainly from in vitro and xenograft studies, while direct clinical relevance in veterinary patients remains largely unproven. The established success of plant-derived anticancer agents such as paclitaxel (from *Taxus brevifolia*), etoposide (from *Podophyllum peltatum* and *P. emodi*), and vincristine (from *Catharanthus roseus*) nevertheless reinforces the broader translational relevance of plant-derived products in oncology.

More broadly, the World Health Organization (WHO) has published a strategy for 2025–2034 calling for the strengthening of the evidence-based use of traditional, complementary and integrative medicine (TCIM), and its safe and effective integration into health systems (Geneva: World Health Organization 2025; Schad et al. 2026). Under a One Health perspective, this broader emphasis on evidence-based evaluation may provide a useful framework for future research on plant extracts and phytochemicals in canine and feline mammary cancer.

Based on the currently available evidence, certain candidates appear more reasonable for prioritization in following studies. Among the isolated phytochemicals, celastrol, HHT and BITC appear particularly promising because they combine relatively strong in vitro activity with mechanistic relevance, while BITC has also exhibited antitumor activity in xenograft models. Among the plant extracts, *Euphorbia royleana* appears to merit particular attention because of its low reported *IC*_*50*_ values and in vivo activity in xenograft models. For studies associated with FMC, initial in vitro evaluation of these candidates may be a reasonable starting point. However, species-specific safety and pharmacokinetic considerations remain essential.

### Clinical caution

From the phytochemicals and plant extracts discussed above, some may merit further investigation, although selection of candidates for future clinical evaluation should not rely exclusively on cytotoxicity or in vivo activity in murine models. Other important aspects include pharmacokinetics, potential toxicity, adverse effects, and drug interactions. For example, homoharringtonine (HHT) exhibited exceptional cytotoxicity and has a strong mechanistic rationale. It is partly metabolized by plasma esterases, and its semi-synthetic analogue omacetaxine mepesuccinate is not expected to exhibit major CYP or P-gp inhibition at clinically used doses in humans (Damlaj et al. 2016; U.S. Food and Drug Administration 2021). However, toxicity has been reported in beagle dogs, and therefore HHT should be approached cautiously. Any further evaluation should include rigorous species-specific safety and pharmacokinetic assessment before consideration in additional companion animal models, including FMC.

Comparative oncology offers opportunities but also complexities. Some molecular pathways affecting FMC overlap with those affecting CMC. However, the two species demonstrate differences, such as distinct pharmacokinetics, that influence the efficacy and safety of different phytochemicals. For example, cats exhibit reduced hepatic glucuronidation, which may significantly alter the metabolism of compounds such as BBR. Further research should follow with standardized experimental designs, detailing pharmacokinetic profiles in both dogs and cats. Controlled clinical trials are necessary to establish therapeutic relevance. In addition, future studies should determine whether selected phytochemicals or standardized plant extracts can be combined with conventional therapies in a safe and biologically meaningful way in dogs and cats with mammary tumors.

## Conclusion

CMTs and FMTs represent a challenge in veterinary oncology because of their high prevalence, the aggressive behavior of malignant and advanced cases, and the limitations of current systemic therapies. Several plant-derived products have been reported to modulate biologically relevant processes. However, most of the currently available evidence remains preclinical and is derived mainly from in vitro and xenograft studies. Thus, direct clinical relevance in veterinary patients remains largely unproven, and plant-derived products should currently be regarded as promising but still experimental candidates in comparative mammary oncology.

Clinical translation requires further research, including pharmacokinetic and safety studies, and clinically relevant combination strategies, as species-specific pharmacokinetics, herb–drug interactions, toxicity, limited bioavailability, and formulation-related issues remain important barriers. These concerns are particularly important when extrapolating data from dogs to cats, as differences in drug metabolism, such as the reduced glucuronidation capacity of cats, may alter the disposition and toxicity of several phytochemicals.

A major gap in the field is the marked imbalance between canine and feline data. Most available studies have been performed in canine models. Despite its aggressive behavior, only one study on FMC cells was identified in this review. To our knowledge, this review is the first to focus specifically on plant extracts and phytochemicals in CMTs and FMTs. By consolidating available evidence, identifying translational and clinical barriers, and acknowledging the research gap in cats, this review may help guide future species-specific preclinical and translational research in veterinary comparative oncology.

## Supplementary Information

Below is the link to the electronic supplementary material.

Supplementary file1 (DOCX 15 KB)

## Data Availability

Data sharing is not applicable to this article as no new data were created or analyzed in this study.

## Abbreviations

ADM: Adriamycin
AhR: Aryl hydrocarbon receptor
Akt: Protein kinase B
AMPK: Adenosine monophosphate-activated kinase
AR: Androgen receptor
ATF4: Activating transcription factor 4
ASPCA: American Society for the Prevention of Cruelty to Animals
BBR: Berberine
BCRP: Breast cancer resistance protein
BCSC: Breast cancer stem cell
BITC: Benzyl isothiocyanate
CBD: Cannabidiol
CDK1: Cyclin-dependent kinase 1
CETSA: Cellular Thermal shift assay
CES1: Carboxylesterase 1
CHOP: C/EBP homologous protein
cIFN-β: Canine interferon-β
CK5/6: Cytokeratin 5/6
CMC: Canine mammary carcinoma
CMT: Canine mammary tumor
COX-2: Cyclooxygenase-2
CSC: Cancer stem cell
CTLA-4: Cytotoxic T-lymphocyte associated protein 4
Curc: Curcumin
CYP: Cytochrome P450
Cyt C: Cytochrome C
DADS: Diallyl disulfide
DIM: Diindolylmethane
ECM: Extracellular matrix
EFSA: European Food Safety Authority
EGFR: Epidermal growth factor receptor
EMA: European Medicines Agency
EMT: Epithelial–mesenchymal transition
ERK: Extracellular signal-regulated kinase
ER: Estrogen receptor
FDA: U.S. Food and Drug Administration
FMC: Feline mammary carcinoma
FMT: Feline mammary tumor
GCV: Ganciclovir
GGT: Glutamyl transpeptidase
Gli1: Glioma-associated oncogene homolog-1
GSH: Glutathione
GST: Glutathione-S-transferase
HBC: Human breast cancer
HDAC: Histone deacetylase
HER2: Human epidermal growth factor receptor
Hh: Hedgehog
HHT: Homoharringtonine
HIF-1α: Hypoxia inducible factor 1α
HSV-tk: HSV-thymidine kinase
I3C: Indole-3-carbinol
*IC*_50_: Half-maximal inhibitory concentration
IL: Interleukin
IMC: Inflammatory mammary carcinoma
IRE1a: Inositol-requiring enzyme 1α
ISO: Isorhamnetin
ITC: Isothiocyanate
JNK: C-Jun N-terminal kinase
MAPK: Mitogen activated protein kinase
MATE1: Multidrug and toxin extrusion protein 1
MC: Metronomic chemotherapy
MEK: Mitogen-activated protein kinase kinase
MG: Methyl gallate
MMP: Matrix metalloproteinase
MRP: Multidrug resistance-associated protein
MT: Matrine
mTOR: Mechanistic target of rapamycin
Nanog: Nanog Homeobox
NF-κB: Nuclear factor kappa-light-chain-enhancer of activated B cells
NLC: Nanostructured lipid carriers
NQO: NAD(P)H:quinone oxidoreductase
NSAID: Non-steroidal anti-inflammatory drug
OATP: Organic anion transporting polypeptides
OCT2: Organic cation transporter 2
Oct4: Octamer-binding transcription factor 4
PCNA: Proliferating cell nuclear antigen
PD-L1: Programmed cell death 1 ligand 1
PERK: Protein kinase R-like endoplasmic reticulum kinase
PGE2: Prostaglandin E2
P-gp: P-glycoprotein
PI3K: Phosphoinositide 3-kinase
PKC-α: Protein Kinase C-α
PKM2: Pyruvate kinase M2
PLK1: Polo-like kinase 1
PLP: Plantain polysaccharide
PLT: Palmatine
PPARγ: Peroxisome proliferator-activated receptor gamma
PR: Progesterone receptor
PTEN: Tumor suppressor phosphatase and tensin homolog
PTX: Paclitaxel
RANK: Receptor Activator of Nuclear Factor-kappa B
RES: Resveratrol
Smad: Suppressor of mothers against decapentaplegic
Sox2: SRY-box transcription factor 2
STAT3: Signal transducer and activator of transcription 3
TGF-β: Transforming growth factor-β
TILs: Tumor infiltrating lymphocytes
TIM-3: T-cell immunoglobulin and mucin-domain containing-3
TME: Tumor microenvironment
TNBC: Triple-negative breast cancer
TNF: Tumor necrosis factor
TSN: Toosendanin
UDPGT: Uridine 5'-diphospho-glucuronosyltransferase
UPR: Unfolded protein response
VEGF: Vascular endothelial growth factor
VISTA: V-domain Ig suppressor of T cell activation
Wnt: Wingless-Type MMTV Integration Site Family
WWP1: WW domain-containing E3 ubiquitin protein ligase 1
XBP1: X-box-binding protein 1
XIAP: X-linked inhibitor of apoptosis
ZER: Zerumbone
α-SMA: α-Smooth muscle actin
