# Supplementary material for: Plant extracts and phytochemicals in canine and feline mammary cancer models: current evidence and comparative perspectives
Source: Vet Res Commun. 2026 Jun 17;50(5):397. doi: 10.1007/s11259-026-11336-8 (PMC13275774; doi:10.1007/s11259-026-11336-8)
Supplement: Supplementary file 1 — Supplementary file1 (DOCX 15 KB) [file 11259_2026_11336_MOESM1_ESM.docx]

**Herbal Products as Adjuvants in Canine and Feline Mammary Tumors: Current Evidence, Comparative Oncology Perspectives, and Future Directions**

**Iason-Spyridon Patergiannakis and Ioannis S. Pappas***

Laboratory of Pharmacology and Toxicology, Faculty of Veterinary Medicine, University of Thessaly, GR-43100 Karditsa, Thessaly, Greece

***Corresponding author:** E-mail address: [ipappas@vet.uth.gr](mailto:ipappas@vet.uth.gr)

Tel: +30 6976601035

Fax: +30 2441066041

E-mail address of each author:

ipatergiannakis@vet.uth.gr

[ipappas@vet.uth.gr](mailto:ipappas@vet.uth.gr)

Supplementary material 1
The detailed queries were:

(((((((((((((((((Canine mammary tumor) OR (Canine Mammary cancer)) OR (Canine breast cancer) ) OR (Canine mammary carcinoma cell line)) OR (CMT-U27)) OR (CMT-U309)) OR (Feline mammary tumor)) OR (Feline breast cancer)) OR (Feline mammary cancer)) OR (Feline breast cancer)) OR (Feline mammary carcinoma cell line)) OR (FCTp)) OR (FMCm)) ) AND ((((Herbal extract) OR (Phytochemical)) OR (natural product)) OR (plant extract)),
which returned 46 results in PubMed and

Canine mammary tumor OR Canine Mammary cancer OR Canine breast cancer OR Canine mammary carcinoma cell line OR CMT-U27 OR CMT-U309 OR Feline mammary tumor OR Feline breast cancer OR Feline mammary cancer OR Feline breast cancer OR Feline mammary carcinoma cell line OR FCTp OR FMCm AND ( Herbal extract OR Phytochemical OR natural product OR plant extract ) 207 results in Scopus.

Additional queries included: *Canine mammary OR Feline mammary AND extract* → 70 PubMed results

*((Mammary Neoplasms, Animal [MeSH Terms]) AND (phytotherapy[MeSH Terms])) AND (plant extract[MeSH Terms])* → 23 PubMed results

*((Mammary Neoplasms, Animal [MeSH Terms]) AND (phytotherapy[MeSH Terms]))* → 32 PubMed results

PubMed searches for “feline mammary” returned 448 results and “canine mammary” 2136 results.
